# Supplementary figures and images for: In heart failure reactivation of RNA-binding proteins is associated with the expression of 1,523 fetal-specific isoforms
Source: PLoS Comput Biol. 2022 Feb 28;18(2):e1009918. doi: 10.1371/journal.pcbi.1009918 (PMC8912908; doi:10.1371/journal.pcbi.1009918)

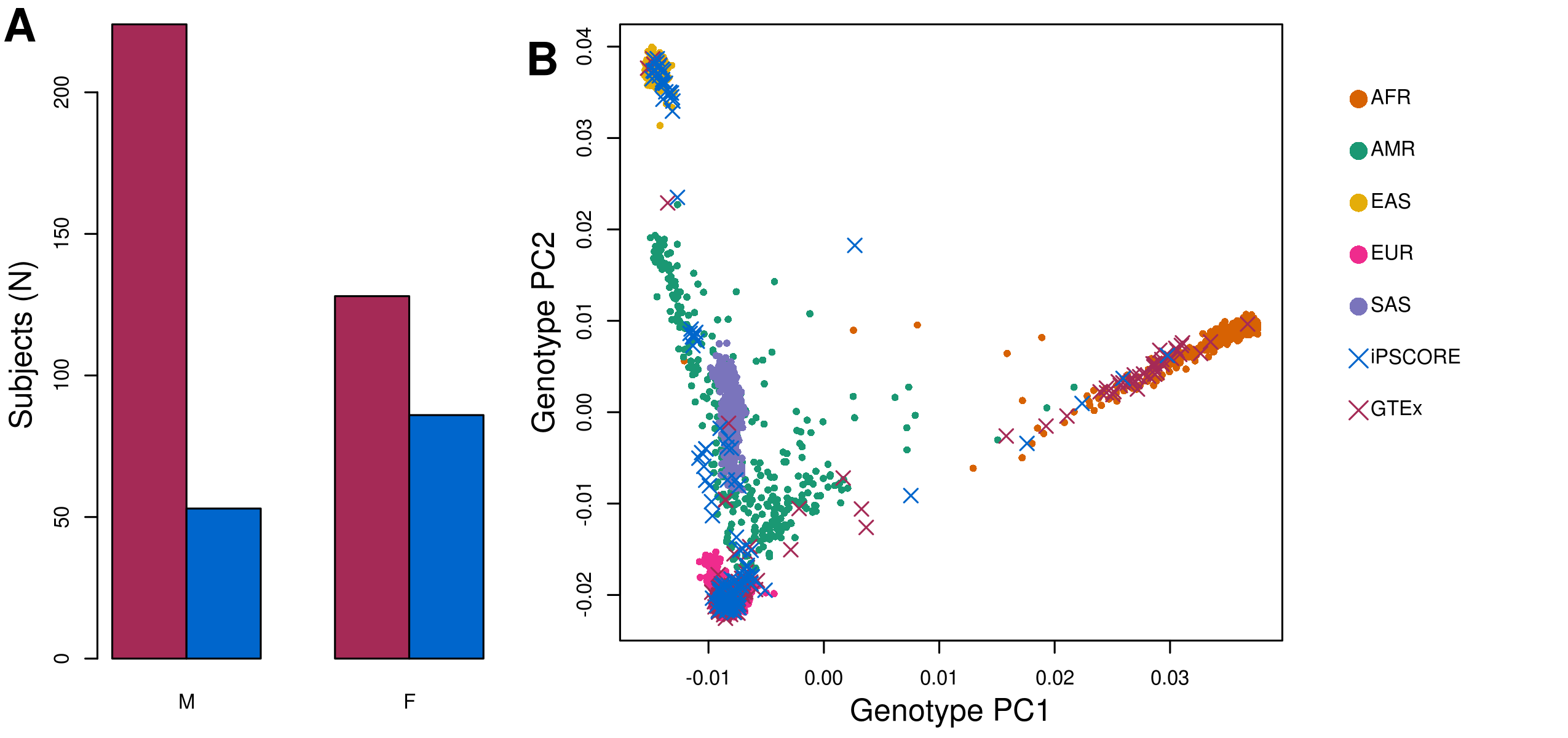

Supplement: S1 Fig — (A) Sex distribution between iPSCORE individuals (blue) and GTEx individuals (maroon). (B) PCA showing the ancestry of all subjects. Colored circles represent ancestry of individuals from the 1000 Genomes Project. GTEx and iPSCORE individuals are represented by pink and blue “X”, respectively. Details are in S1 Table. (TIFF) [file pcbi.1009918.s001.tiff]

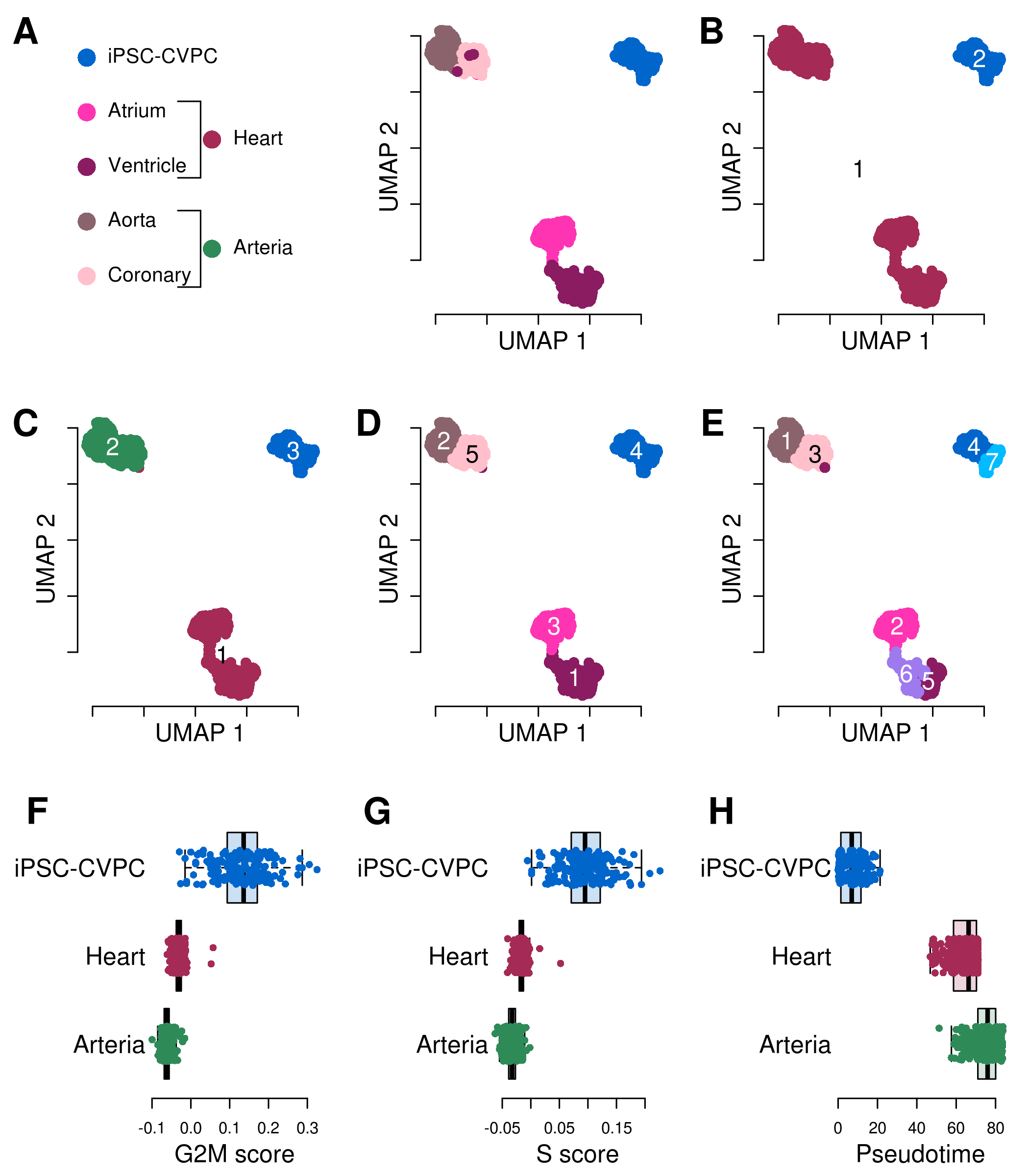

Supplement: S2 Fig — (A-E) UMAP plots showing how the 966 bulk RNA-seq CVS samples cluster at varying resolutions (S3 Fig). Panel (A) shows the samples according to their tissue of origin (iPSC-CVPC, adult atrium, ventricle, aorta or coronary artery). In panels (B-E) samples are colored based on clustering (B) at low resolution (resolution = 0); (C) at intermediate-low (resolution = 0.01); (D) at intermediate-high resolution (resolution = 0.2); and (E) at high resolution (resolution = 0.7). Low resolution separated the samples based on their developmental stage (iPSC-CVPC and adult tissues); intermediate-low resolution divided the samples into three clusters corresponding to iPSC-CVPC, adult heart (atrial appendage and left ventricle samples) and adult arteria (aorta and coronary artery samples); intermediate-high resolution separated the samples into the five CVS tissues (iPSC-CVPC, adult atrium, ventricle, aorta or coronary artery); and high resolution separated samples derived from the same tissue-type suggesting the presence of cellular heterogeneity across samples. To increase statistical power, for all downstream analyses we used the clusters obtained at intermediate-low resolution (i.e., we combined atrium and ventricle samples into one group referred to as “adult heart”, and aorta and coronary artery into one group referred to as “adult arteria”). There were 5 adult heart samples that cluster with the adult arteria and 1 adult arteria sample that clusters with the adult heart. Most likely, their cell type composition differs from the other samples of the same label due to the area in which they were collected. We retained the original labeling of the 5 samples as adult heart and the 1 sample as adult arteria for downstream analyses. (F-H) Boxplots showing (F) G2M score, (G) S phase score and (H) pseudotime calculated using Seurat or Monocle. iPSC-CVPC display a higher G2M score compare with both heart and arteria (heart: p = 3.1 x 10^−83^, arteria: p = 1.7 x 10^−79^, Mann-Whitney U [file pcbi.1009918.s002.tiff]

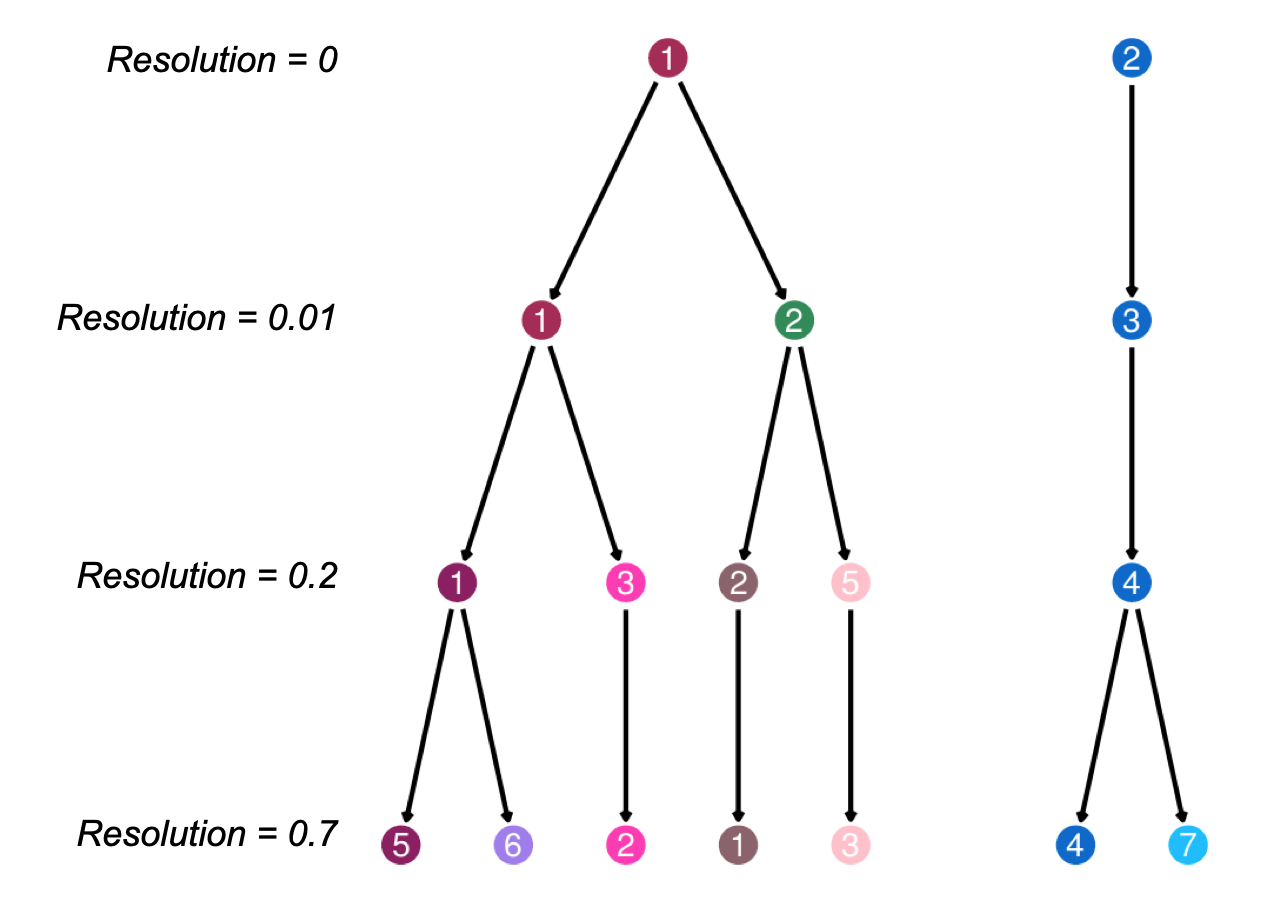

Supplement: S3 Fig — Cluster trees showing how 966 RNA-seq samples are grouped together with KNN clustering at different resolutions. The nodes are color-coded in the same manner as the clusters in the companion UMAP plots in S2A–S2E Fig. Lower resolution separates the samples based on developmental stage, while intermediate and higher resolutions resolve iPSC-CVPC (right) and adult CVS (left) samples into sub-clusters. (TIF) [file pcbi.1009918.s003.tif]

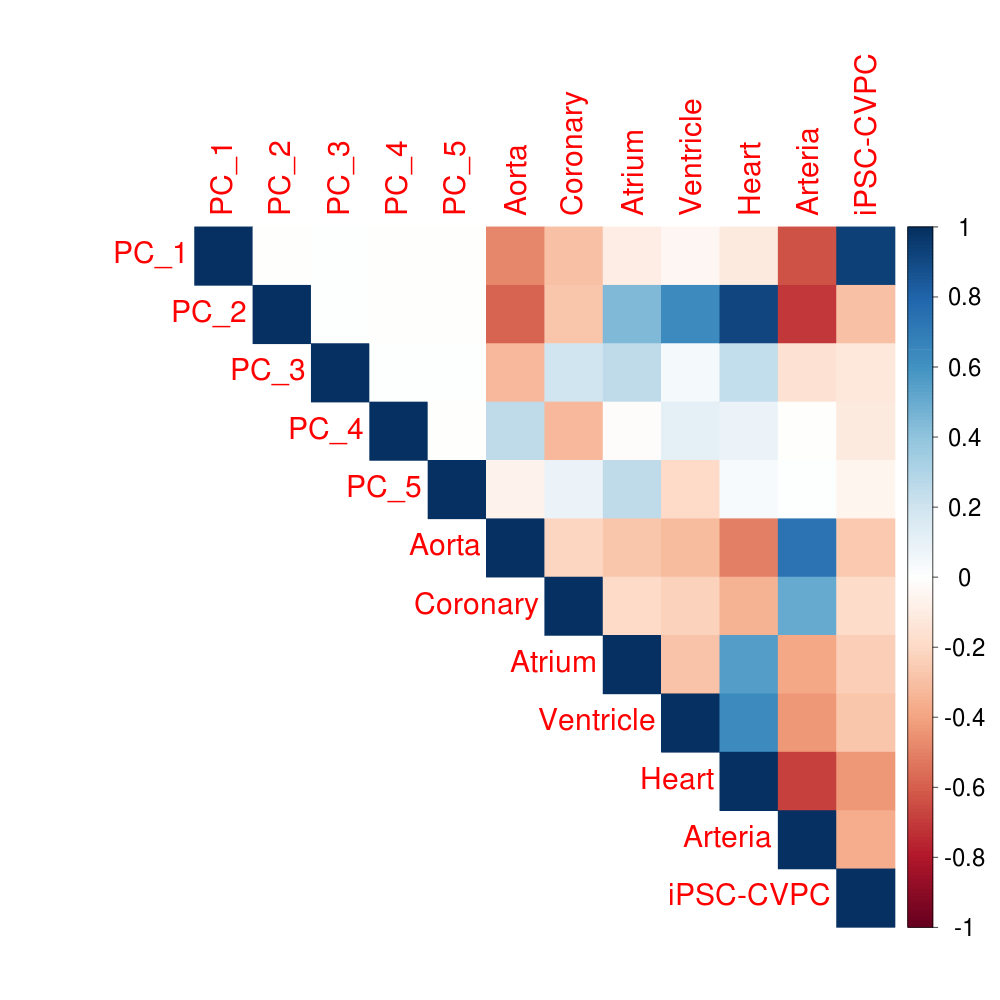

Supplement: S4 Fig — Corrplot [101] showing the Pearson correlation between the top 5 principal components calculated on the 966 CVS bulk RNA-seq samples and binary vectors indicating the type of tissue—aorta, coronary, atrium, ventricle, heart (atrium and ventricle), arteria (aorta and coronary), and iPSC-CVPC (S2 Table). The legend indicates the strength of the correlation for each pairwise comparison with blue representing a positive correlation and red representing a negative correlation. This correlation analysis shows that that the top principal component (PC1, 32.2% variance explained) is strongly associated with CVS developmental stage, whereas PC2 (19.4% variance explained) divides the adult CVS samples between heart and arteria. These results confirm that the two adult heart tissues (atrium and ventricle), as well as the two adult arteria tissues (aorta and coronary) have similar transcriptomes and supports combining samples in each of the three clusters obtained at intermediate-low resolution (S2 and S3 Figs) to increase power. The results also show that the two adult CVS samples are more similar to each other than to the fetal-like iPSC-CVPC. (TIF) [file pcbi.1009918.s004.tif]

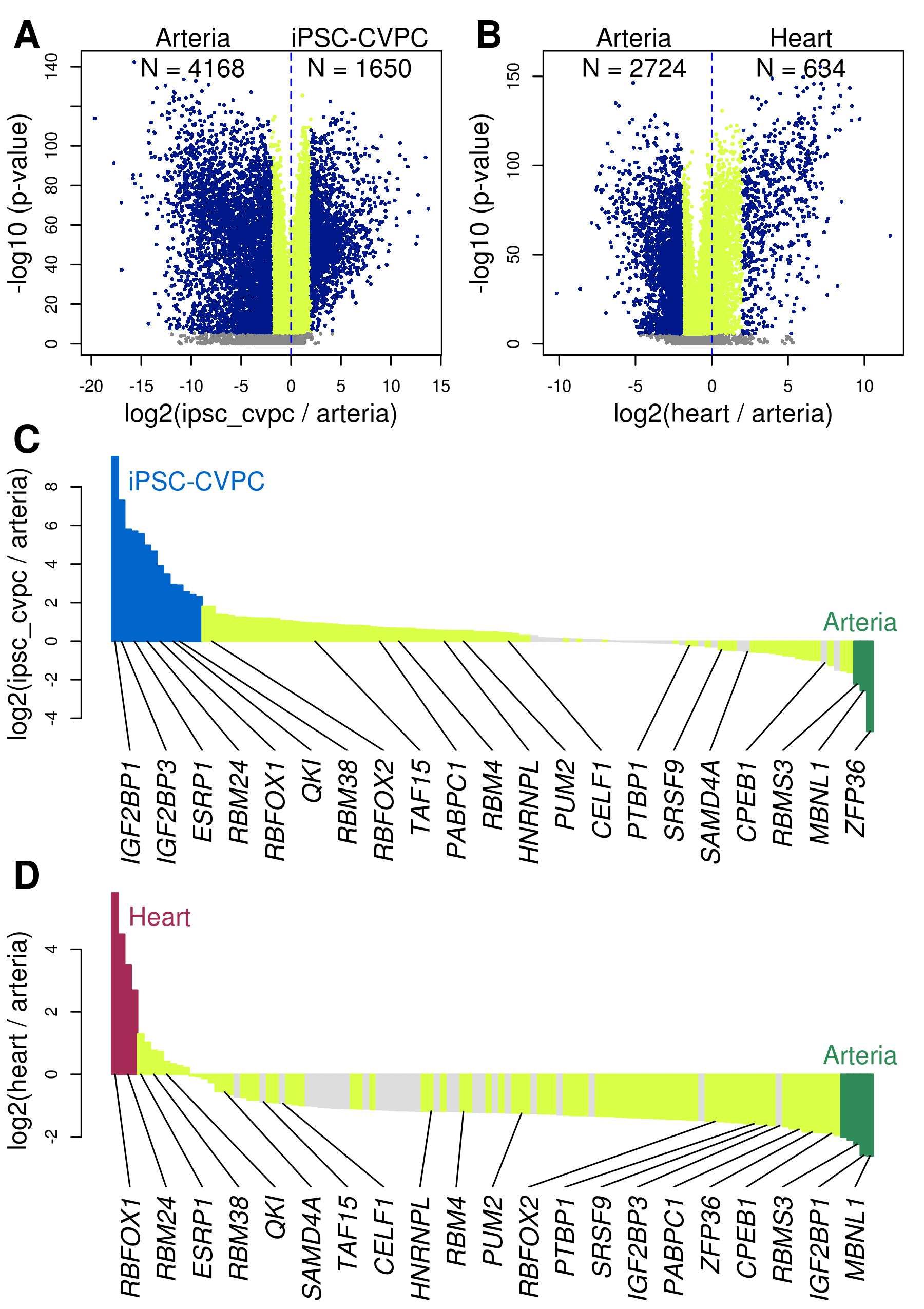

Supplement: S5 Fig — (A, B) Volcano plots showing the differentially expressed genes between (A) iPSC-CVPC and adult arteria, and (B) adult heart and adult arteria. Genes that are not differentially expressed are shown in gray, differentially expressed genes with log2 ratio between -2 and 2 are in yellow, and genes with CVS tissue-specific expression (i.e. at least four-fold expression difference; log2 ratio > 2 or < -2) are shown in blue. (C, D) Barplots showing the log2 ratio between the mean expression in (C) iPSC-CVPC and adult arteria and (D) adult heart and adult arteria for 122 RBPs with a known motif [31–33]. RBPs shown in blue, maroon and green are iPSC-CVPC-specific, adult heart-specific and adult arteria-specific, respectively. All other differentially expressed RBPs (FDR < 0.05) are shown in yellow. These plots, in combination with Fig 1D, show that iPSC-CVPC have more overexpressed and specific RBPs than either adult heart or adult arteria, and while there is a greater number of overexpressed RBPs in adult arteria compared with adult heart, the two CVS tissues have similar numbers of tissue-specific RBPs expressed. (TIFF) [file pcbi.1009918.s005.tiff]

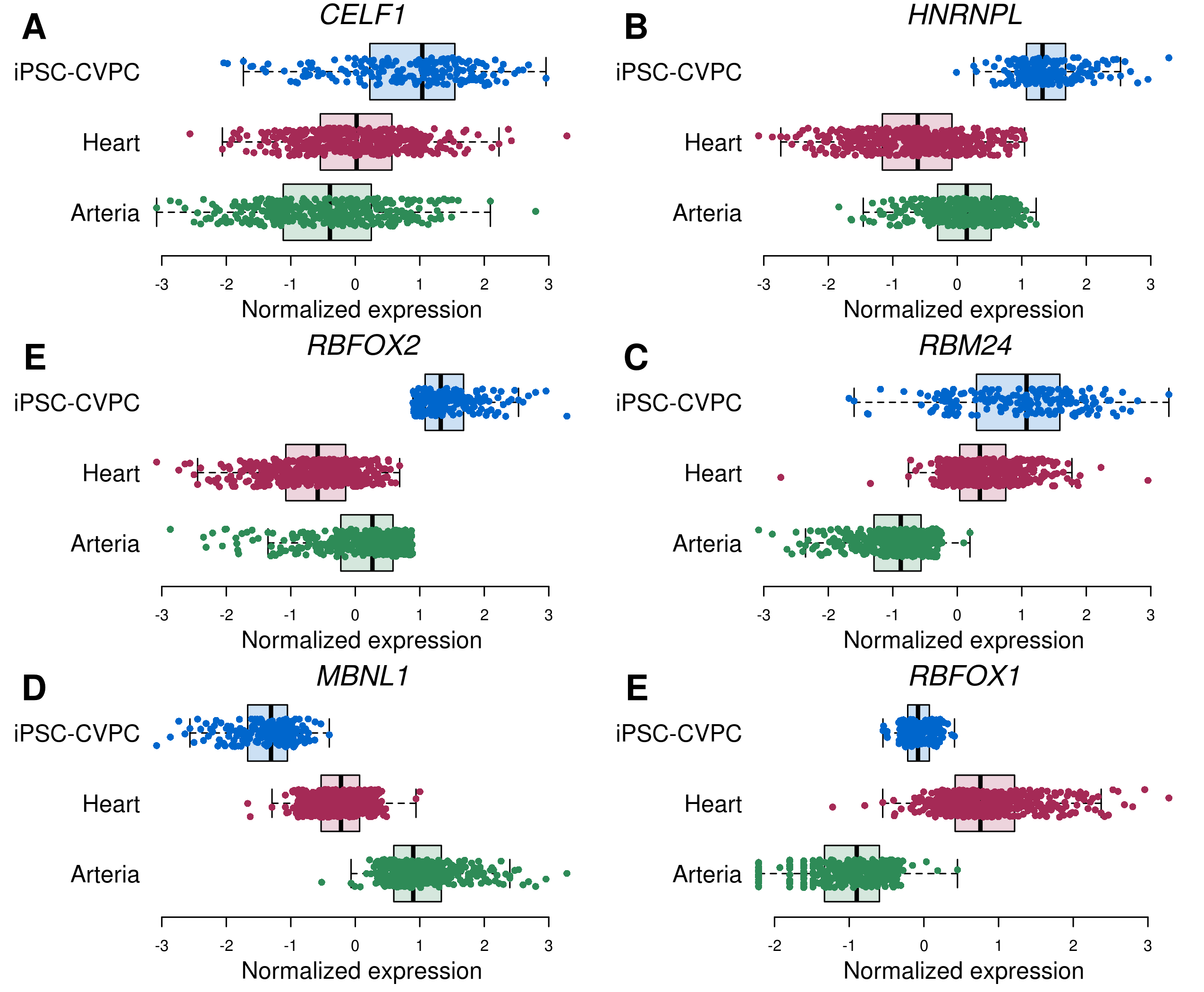

Supplement: S6 Fig — Boxplots showing gene expression levels (TPM) in iPSC-CVPC, adult heart and adult arteria for six RBPs involved in cardiac and muscle development. Four of these genes (CELF1, HNRNPL, RBFOX2 and RBM24) have known functions during cardiac fetal development and were overexpressed in iPSC-CVPC compared with the two adult tissues, whereas the other two (MBNL1 and RBFOX1) have adult-specific functions and were expressed at higher levels in adult arteria and adult heart, respectively, compared with iPSC-CVPC. (TIFF) [file pcbi.1009918.s006.tiff]

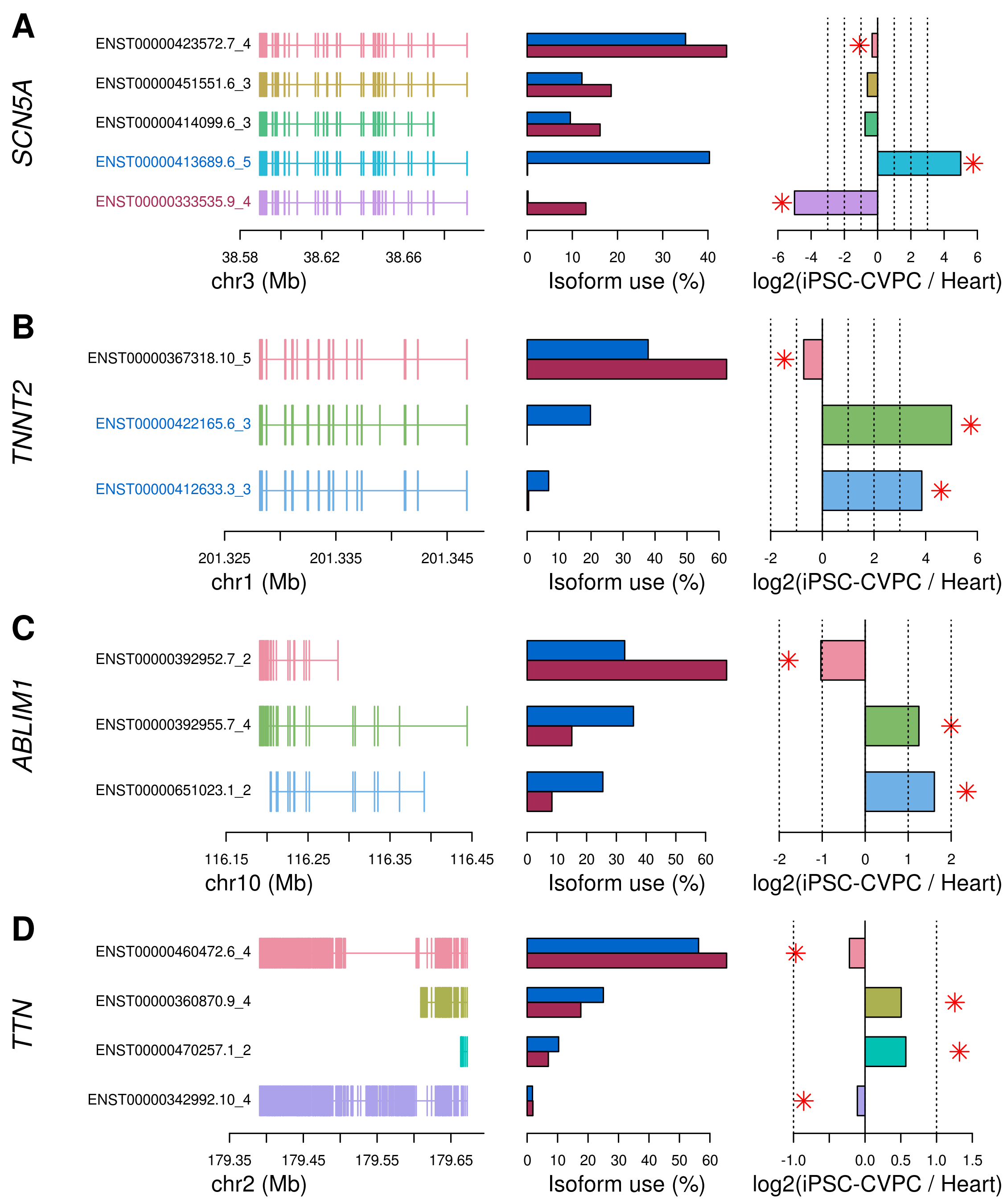

Supplement: S7 Fig — The figure shows the differential isoform usage between iPSC-CVPC (blue in the central panel) and adult heart (purple) for four cardiac genes known to have well-established developmental stage-specific isoforms: SCN5A, TNNT2, ABLIM1 and TTN. Left side of the figure represents the exon structure of each expressed isoform; the barplots in the middle show the mean isoform use (%) of each isoform in iPSC-CVPC (blue) and adult heart (atrium and ventricle, purple). Barplots on the right show the log2 ratio between the mean isoform use in iPSC-CVPC and adult heart. Red stars indicate significantly differentially expressed isoforms (FDR < 0.05). Dotted lines represent one-, two- and four-fold differences between iPSC-CVPC and adult. We found that three SCN5A isoforms, three TNNT2 isoforms, three ABLIM1 isoforms and four TTN isoforms were differentially expressed. Four of these isoforms (two for SCN5A and two for TNNT2) were tissue-specific, having at least a four-fold change difference between CVS tissues. To test if the isoforms expressed in the fetal-like iPSC-CVPC and in the adult heart reflect the known differences between fetal and adult, we further investigated their structure. The main differences between SCN5A fetal and adult isoforms are two mutually exclusive exons that encode for the voltage-sensing transmembrane domain and result in different electrophysiological properties: 6A (fetal) and 6B (adult) [102–104]. We confirmed that they were correctly expressed in the iPSC-CVPC- and adult-specific isoforms (S7A Fig). TNNT2 isoforms differ at the 5’ end, with the longer isoforms being fetal-specific and the shorter isoforms being expressed in the adult [66]. Of the three differentially expressed isoforms, we observed that the two longer isoforms were iPSC-CVPC-specific, whereas the shorter was enriched in the adult heart (S7B Fig). ABLIM1 has four C-terminal LIM domains that play a role in its binding to actin [26]. The shorter isoforms, which only contains three LI [file pcbi.1009918.s007.tiff]

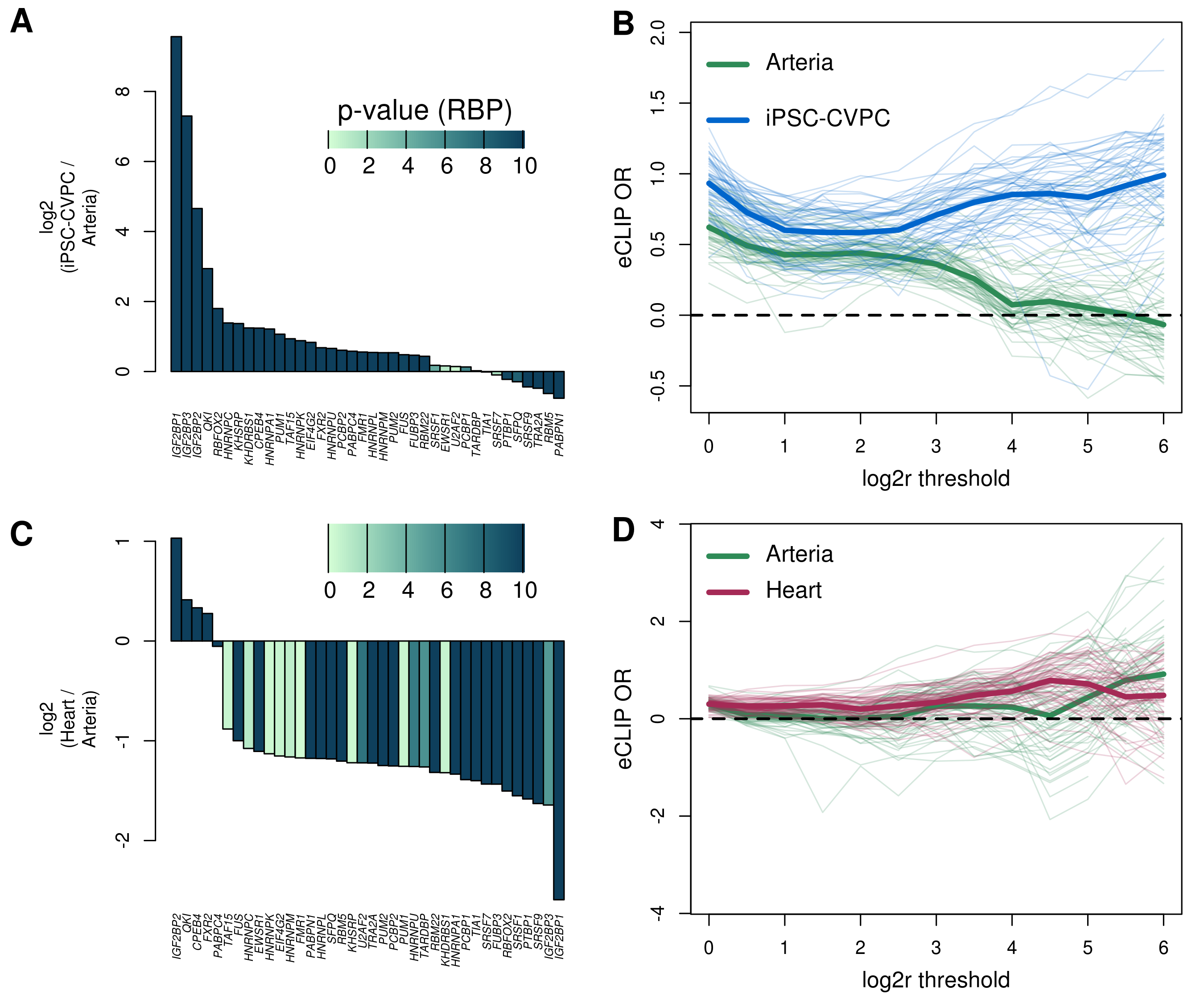

Supplement: S8 Fig — (A, C) Barplots showing the differential expression of 38 RBPs with experimentally determined (eCLIP) binding sites between (A) iPSC-CVPC and adult arteria and (C) adult heart and adult arteria. Colors represent–log10 p-values (S3 Table). Each row represents one RBP. (B, D) Plots showing the enrichment of genomic loci encoding (B) iPSC-CVPC-specific isoforms versus adult arteria-specific isoforms and (D) adult heart-specific isoforms versus adult arteria-specific isoforms for overlapping experimentally determined (eCLIP) RBP binding sites. Each line corresponds to an eCLIP experiment. The X axis corresponds to log2 ratio thresholds: (B) mean isoform usage in iPSC-CVPC/ mean isoform usage in adult arteria; and (D) mean isoform usage in adult heart/mean isoform usage in adult arteria. Enrichment was calculated by comparing the proportion of genes with differentially expressed isoforms passing the log2 ratio thresholds described on the X axis (S5 Table) and overlapping eCLIP peaks against the proportion of genes without any differentially expressed isoforms (FDR > 0.05 for all the isoforms) overlapping eCLIP peaks. The thick lines represent the mean enrichments across all RBP experiments for iPSC-CVPC-specific isoforms (blue), adult heart-specific isoforms (purple) or adult arteria-specific isoforms (green). These plots show that genes with iPSC-CVPC-specific isoforms are more likely to overlap RBP binding sites than genes with adult arteria-specific isoforms; whereas adult heart-specific isoforms and adult arteria-specific isoforms are equally likely to overlap RBP binding sites. (TIFF) [file pcbi.1009918.s008.tiff]

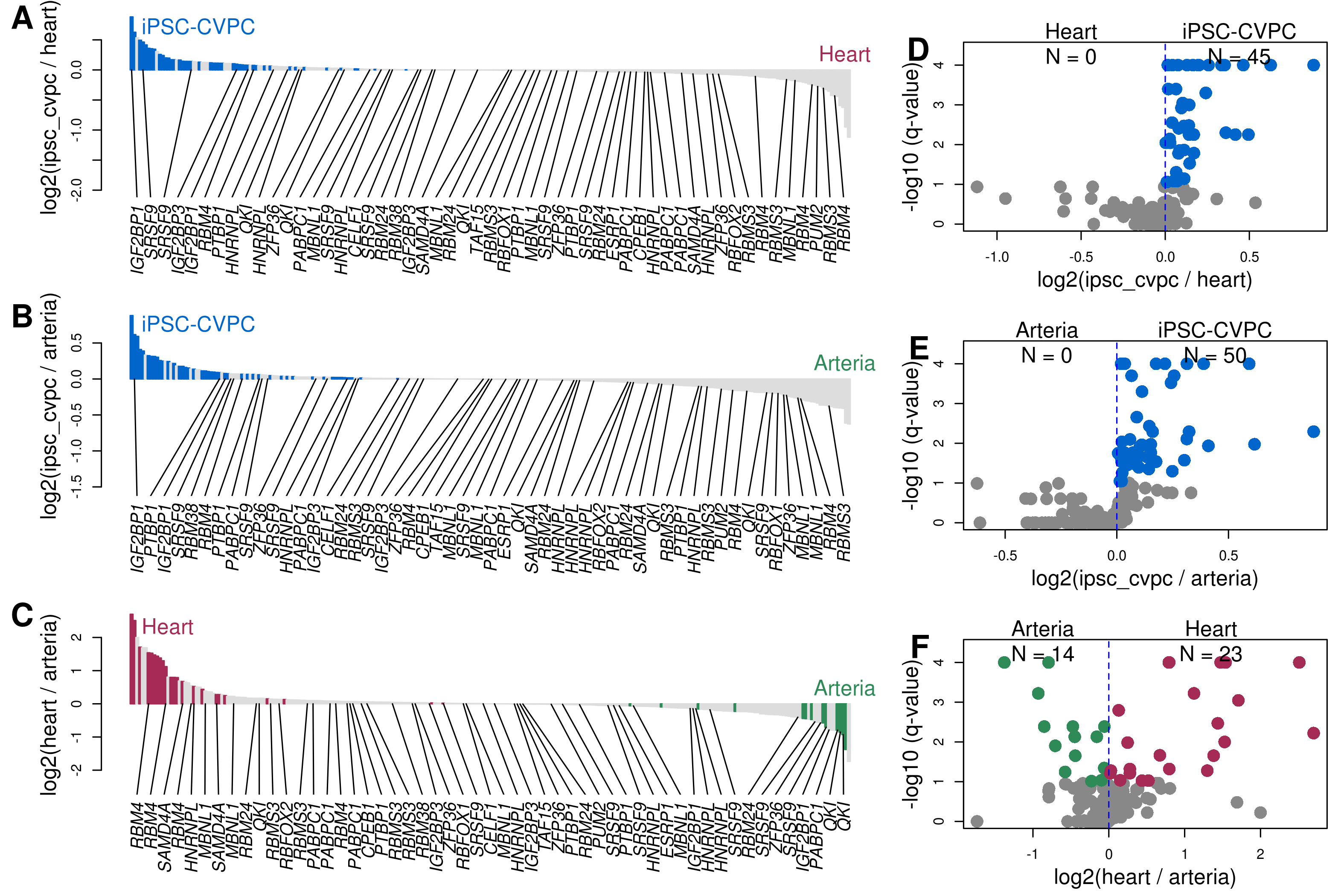

Supplement: S9 Fig — Shown are the differences in the likelihood of the 100 bp upstream of the acceptor splice sites of iPSC-CVPC-specific, adult heart-specific and adult arteria-specific exons to harbor motifs of 122 RBPs. (A-C) Barplots showing the log2 ratio of the likelihood of each CVS tissue-specific splice site to harbor a RBP motif (iPSC-CVPC = blue, adult heart = red, adult arteria = green). (D-F) Volcano plots show the enrichment for each RBP (X axis, same as panels A-C) and its associated -log10(q-value) calculated using Homer findMotifsGenome.pl [98]. Q-values < 1.0 x 10^−5^ are shown as 1.0 x 10^−5^. Colors are as shown in panels A-C. (TIFF) [file pcbi.1009918.s009.tiff]

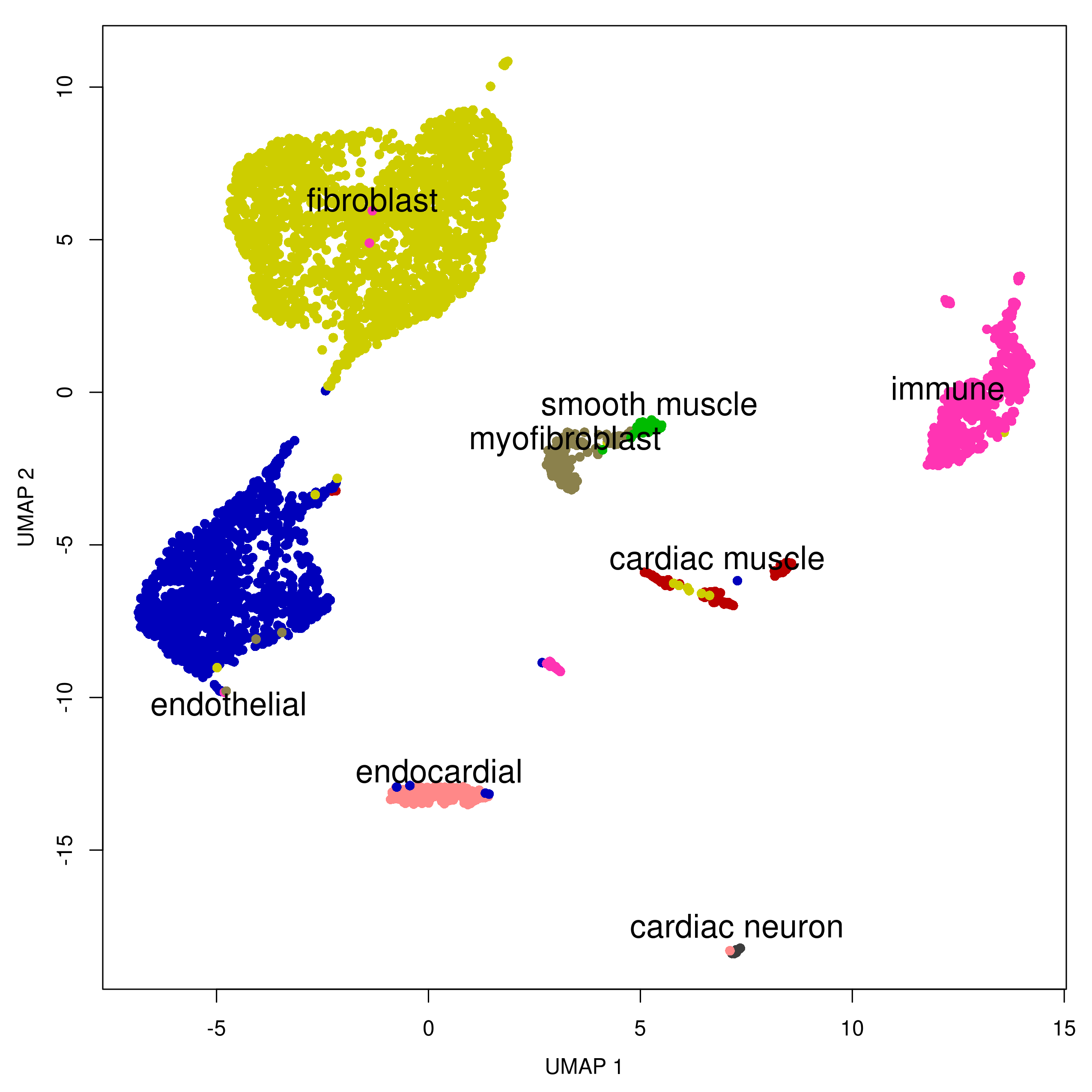

Supplement: S10 Fig — UMAP plot showing the clustering and cell types associated with each of the 4,365 cells obtained from Tabula Muris. Cell labels are as defined by the Tabula Muris Consortium [50]. (TIFF) [file pcbi.1009918.s010.tiff]

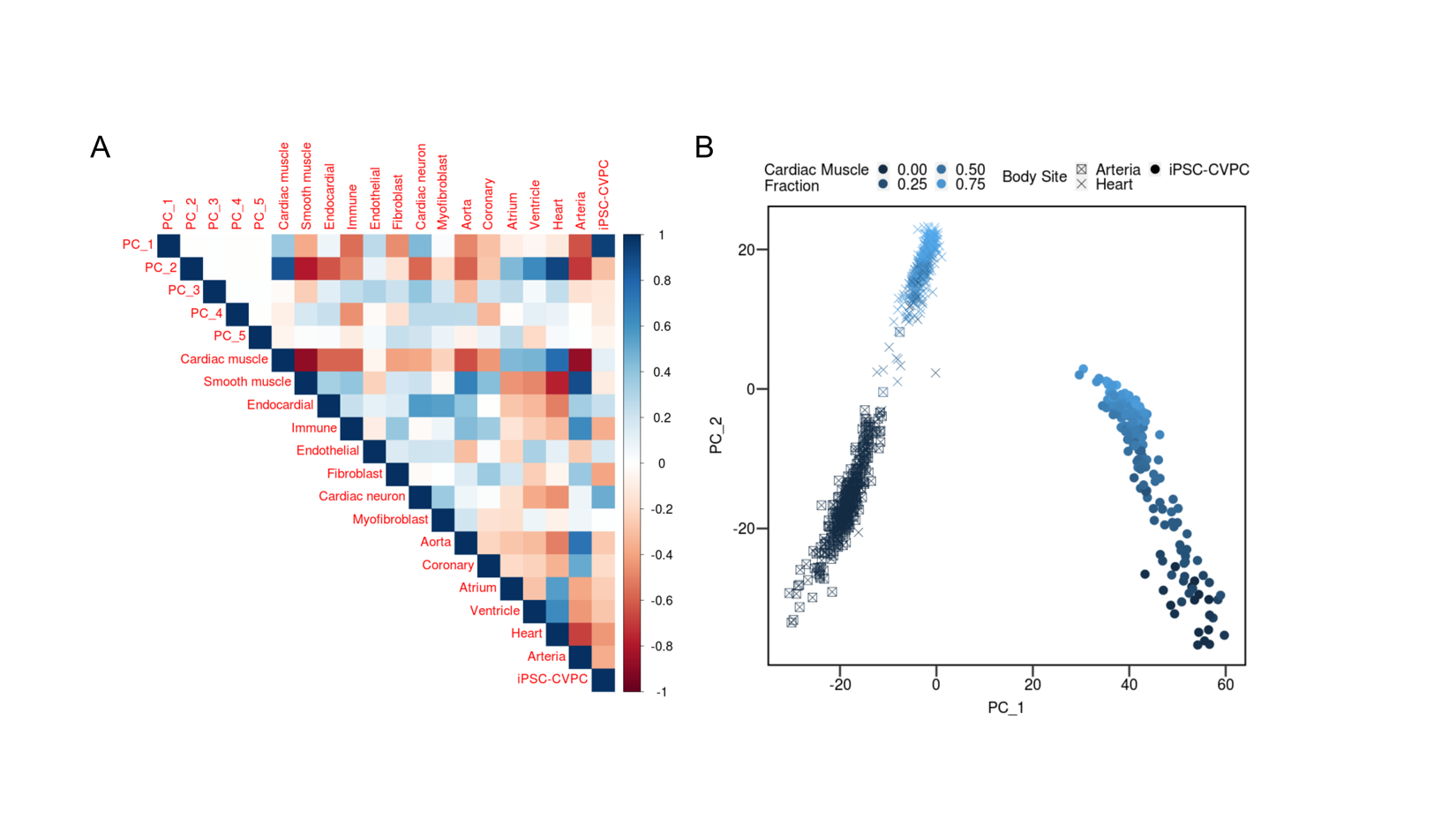

Supplement: S11 Fig — (A) Heatmap showing the Pearson correlations between the top 5 principal components calculated on 2,000 most variable genes across the 966 bulk RNA-seq CVS samples, cell type proportion, and the tissue types of the 966 CVS samples. The top two principal components were correlated with developmental stage (R = 0.934) and cardiac muscle proportion (R = 0.864), respectively, indicating that gene expression levels are strongly associated with cellular heterogeneity and suggesting that the heterogeneity observed at high clustering resolution is likely a consequence of the variability in cell type proportions within each tissue. (B) Scatter plot showing the separation of fetal iPSC-CVPC (right) and adult heart and adult arteria (left) by the first principal component and the separation of samples with larger fraction of cardiac muscle (top/light blue) and those with smaller fraction (bottom/dark blue) by the second principal component. These results further highlight that developmental stage and cellular heterogeneity explain the most variability in the transcriptomes of the 966 CVS samples. (TIF) [file pcbi.1009918.s011.tif]

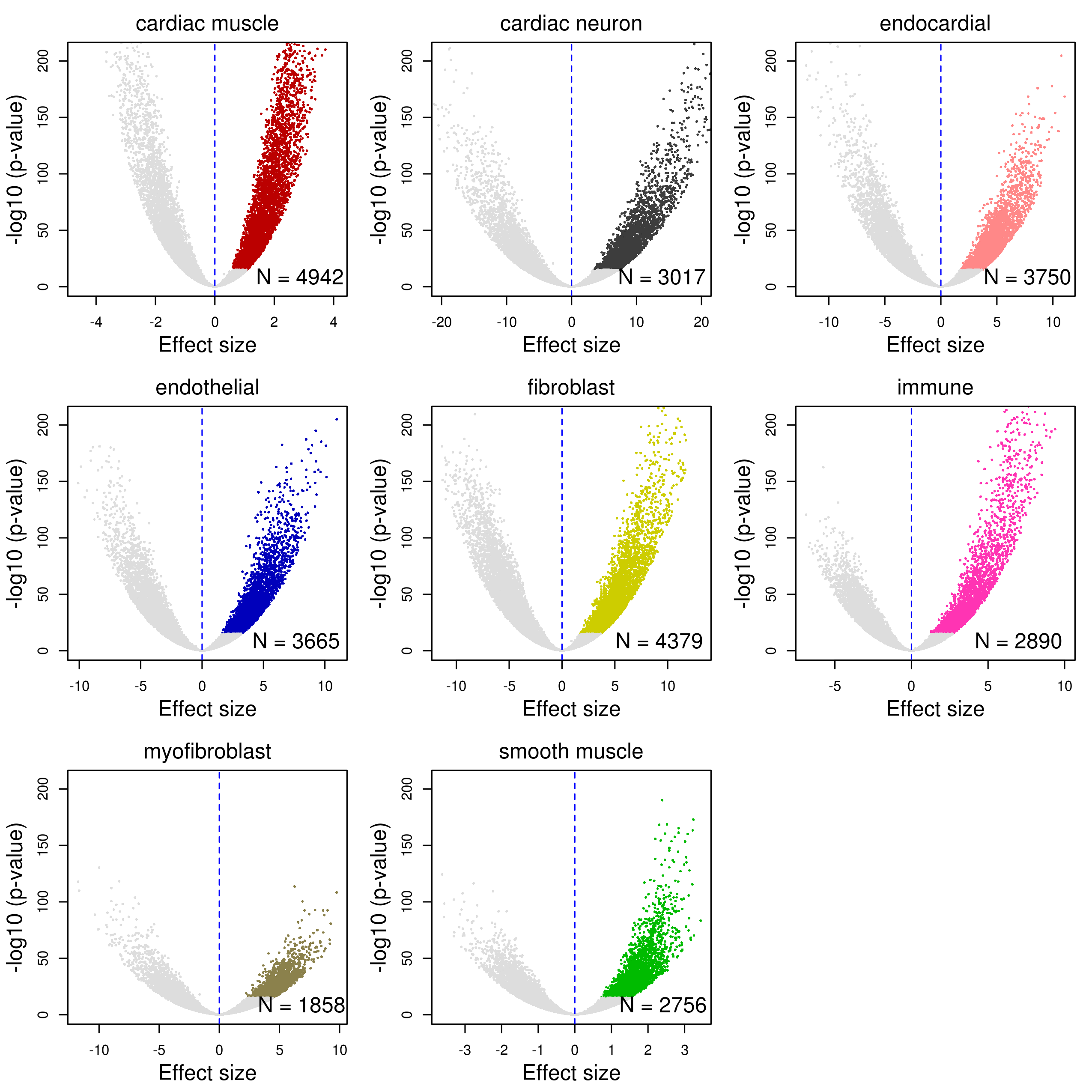

Supplement: S12 Fig — Volcano plots showing the number of genes whose expression is associated with the proportion of indicated cell type. This analysis was performed on all 966 CVS samples using linear regression (lm function in R) between cell type proportions and gene expression levels. X axis represents the effect size and the Y axis represents p-values. All non-significant genes are colored in light gray (FDR > 0.05). Genes significantly associated with each cell type (FDR < 0.05 and effect size > 0) are colored and their number is reported at the bottom-right of each plot. (TIFF) [file pcbi.1009918.s012.tiff]

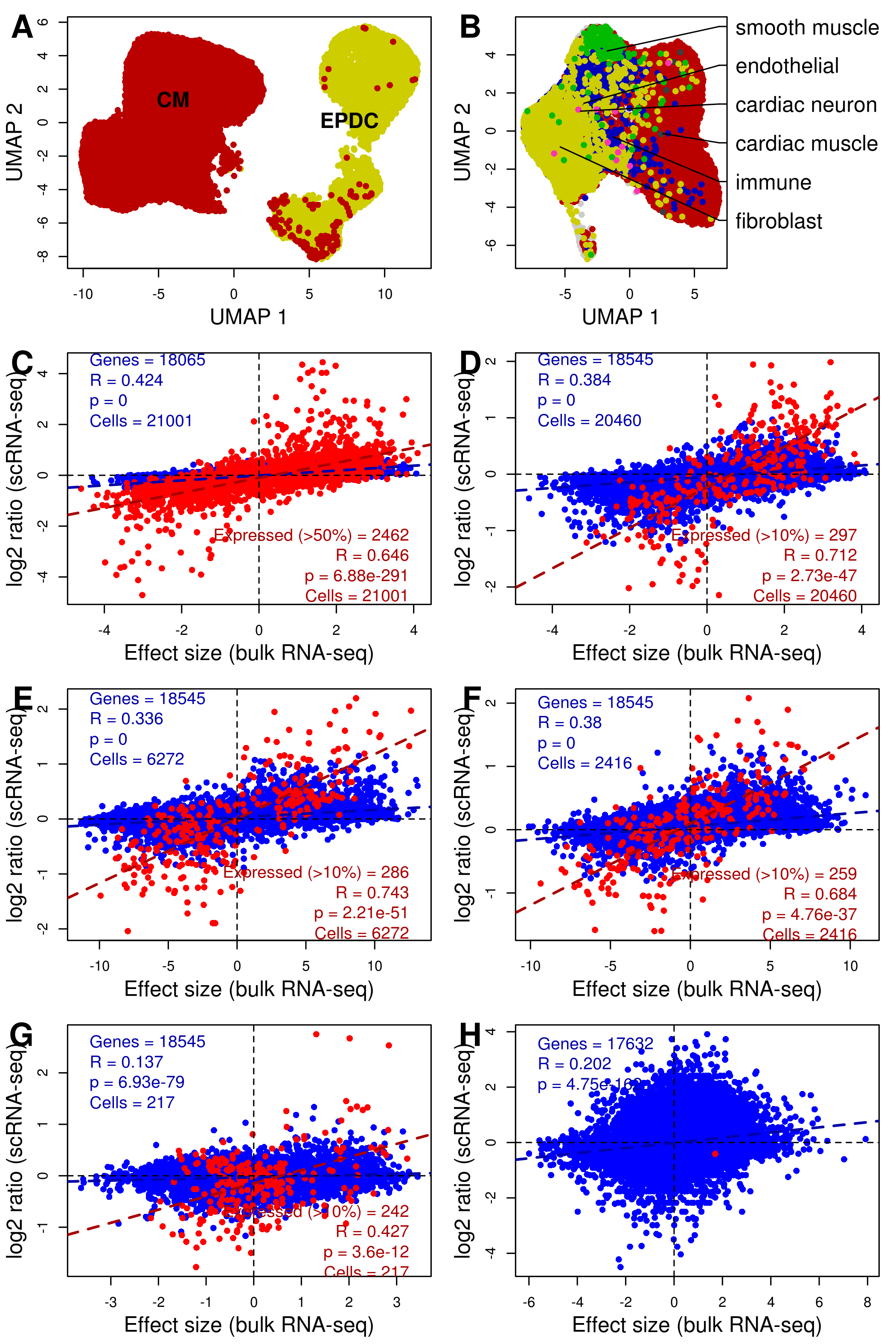

Supplement: S13 Fig — We used two scRNA-seq datasets from iPSC-CVPC and adult heart [14,52] to further characterize the cell type proportions estimated by CIBERSORT. Using scRNA-seq data, we performed differential expression analysis for each cell type and found that the ratio between the expression levels of each gene in a cell type compared with all the other cells was strongly correlated with the effect size of the associations between estimated cell type proportions determined by CIBERSORT deconvolution and gene expression across all cell types. (A-B) Plots showing the UMAP coordinates of (A) 32,026 iPSC-CVPC cells from eight iPSCORE samples (CM: cardiomyocytes; EPDC: epicardial-derived cells); and (B) 33,050 adult left ventricle cells. (C) Scatterplot showing the effect size (linear regression) of the association between gene expression and % of cardiac muscle in the 966 deconvoluted RNA-seq CVS samples (X axis) and the log2 ratio between the expression in CMs and EPDCs in the 32,026 iPSC-CVPC cells (Y axis). (D-G) Scatterplots showing the effect size of the association between gene expression and % of (D) cardiac muscle, (E) fibroblast, (F) endothelial and (G) smooth muscle in the 966 deconvoluted RNA-seq CVS samples (X axis) and the log2 ratio between gene expression in the indicated cell type and all other cells for the 33,050 adult left ventricle cells. Red shows highly expressed genes (expressed in at least 50% of iPSC-CVPC cells or 10% of adult cells). All the lowly expressed genes are shown in blue. Red and blue dashed lines are the regression lines for highly expressed genes and across all tested genes, respectively. In the bottom right corner of each plot, the correlation for the highly expressed genes is shown. In the top left corner, the correlation calculated across all the genes is shown. These plots show that the associations between gene expression and the estimated cell type proportion in the bulk RNA-seq data are significantly strongly correlated with the expression [file pcbi.1009918.s013.tiff]

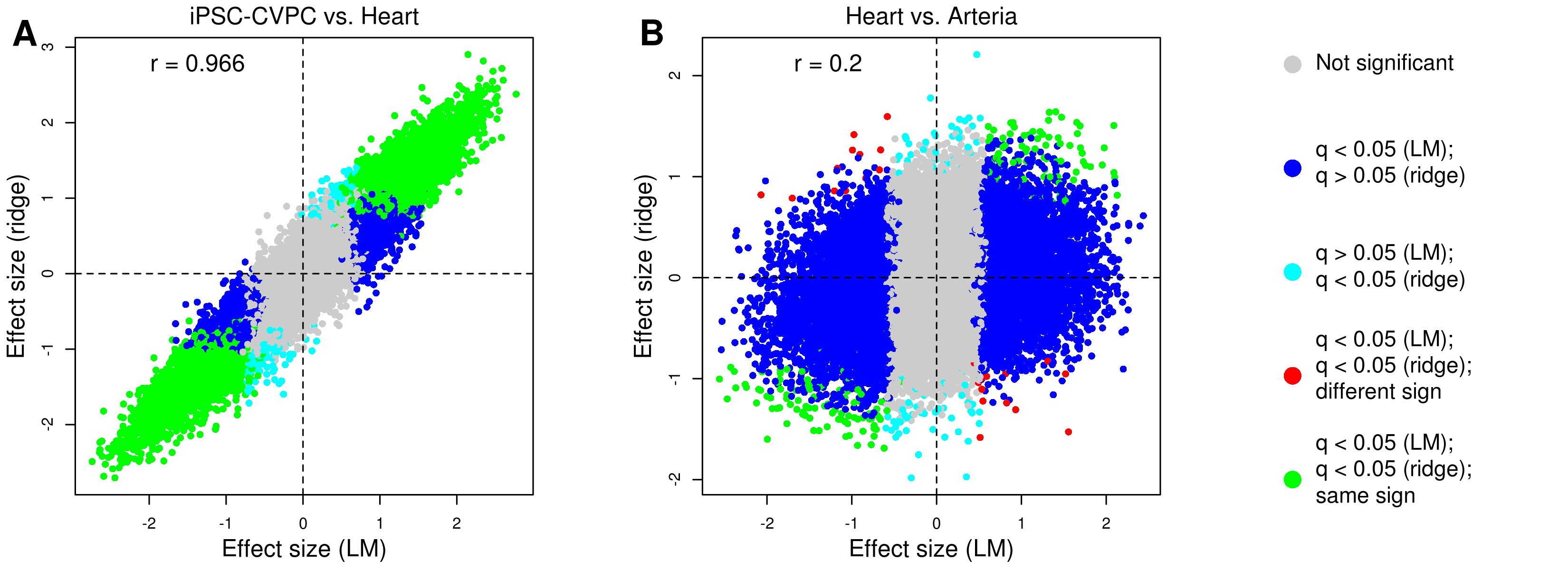

Supplement: S14 Fig — Scatterplots showing the differential isoform usage effect size without considering cell types (linear model, LM, X axis) and considering the cell type proportions as covariates (ridge regression, Y axis) for each pairwise comparison between CVS tissues: (A) iPSC-CVPC vs. adult heart and (B) adult heart vs. adult arteria. Isoforms differentially expressed in both analyses and that have the same effect size sign are shown in green; isoforms whose effect size has different signs between the LM and ridge regression are in red; isoforms that are differentially expressed only using the LM are shown in blue; and isoforms that are differentially expressed only when taking cell type proportions into account (ridge) are in cyan. (TIFF) [file pcbi.1009918.s014.tiff]

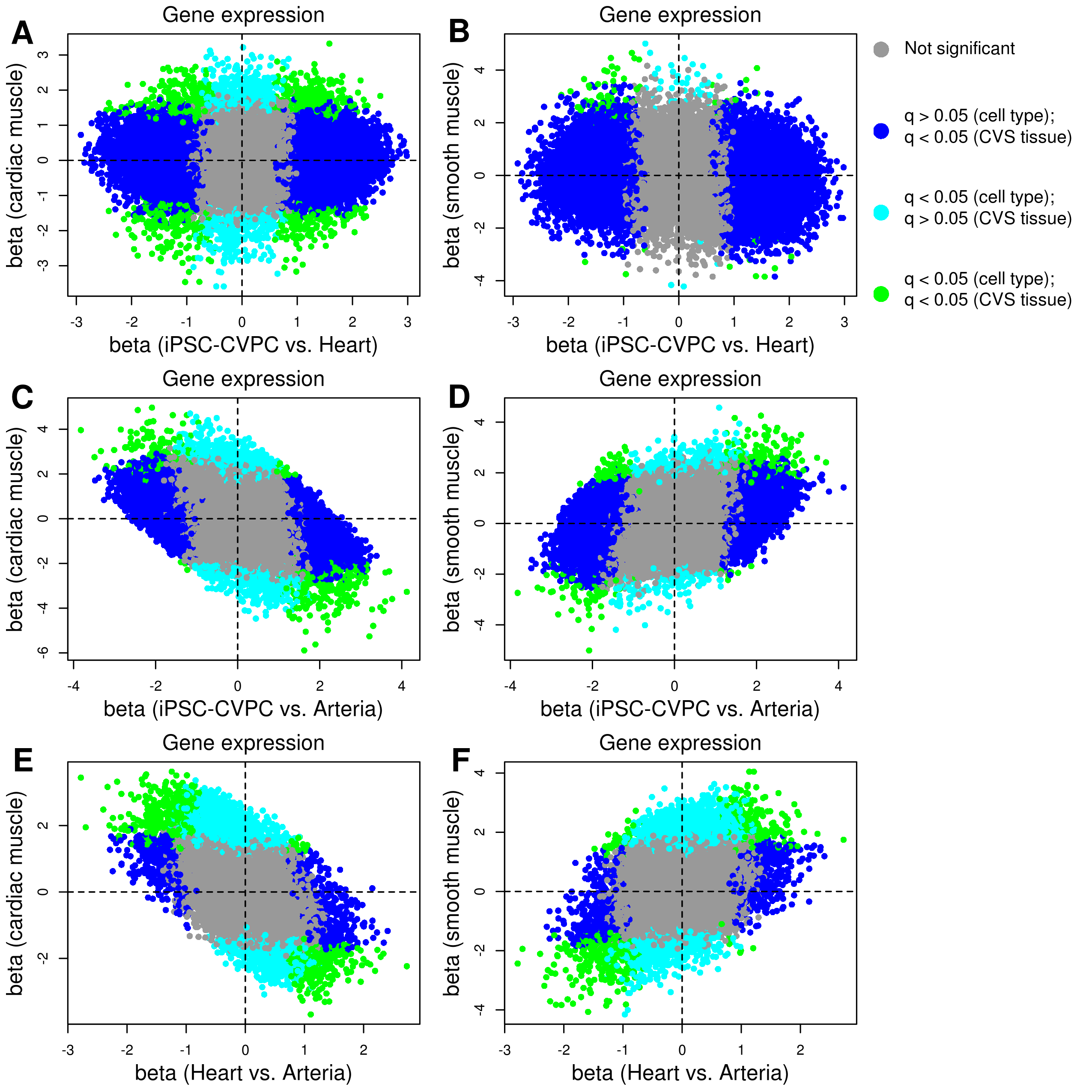

Supplement: S15 Fig — Scatterplots showing differential gene expression effect size between each indicated pair of CVS tissues and the effect size of the association between gene expression and cell type proportion of cardiac muscle or smooth muscle cells. Genes whose expression are not associated with either CVS tissue or cell type proportion are shown in gray; genes significantly associated with both CVS tissue and cell type proportion are in green; genes associated only with cell type are in blue; and genes that are associated only with CVS tissue are in cyan. Overall, we observed a weak correlation between the effect size of the differential expression analysis between iPSC-CVPC and adult and the effect size of the association between gene expression and cardiac muscle proportion (R2 = 0.00925, panel A). This correlation was stronger when comparing the effect size of the differential expression analysis against arteria (R2 = 0.142 and R2 = 0.383, respectively for iPSC-CVPC and adult heart, panels C, E), suggesting that, differences in cell type proportions drive the transcriptomic differences between iPSC-CVPC and adult arteria and between adult heart and adult arteria. Conversely, the differences that we observe between iPSC-CVPC and adult heart are likely due to changes in gene expression within the same cell type. (TIFF) [file pcbi.1009918.s015.tiff]

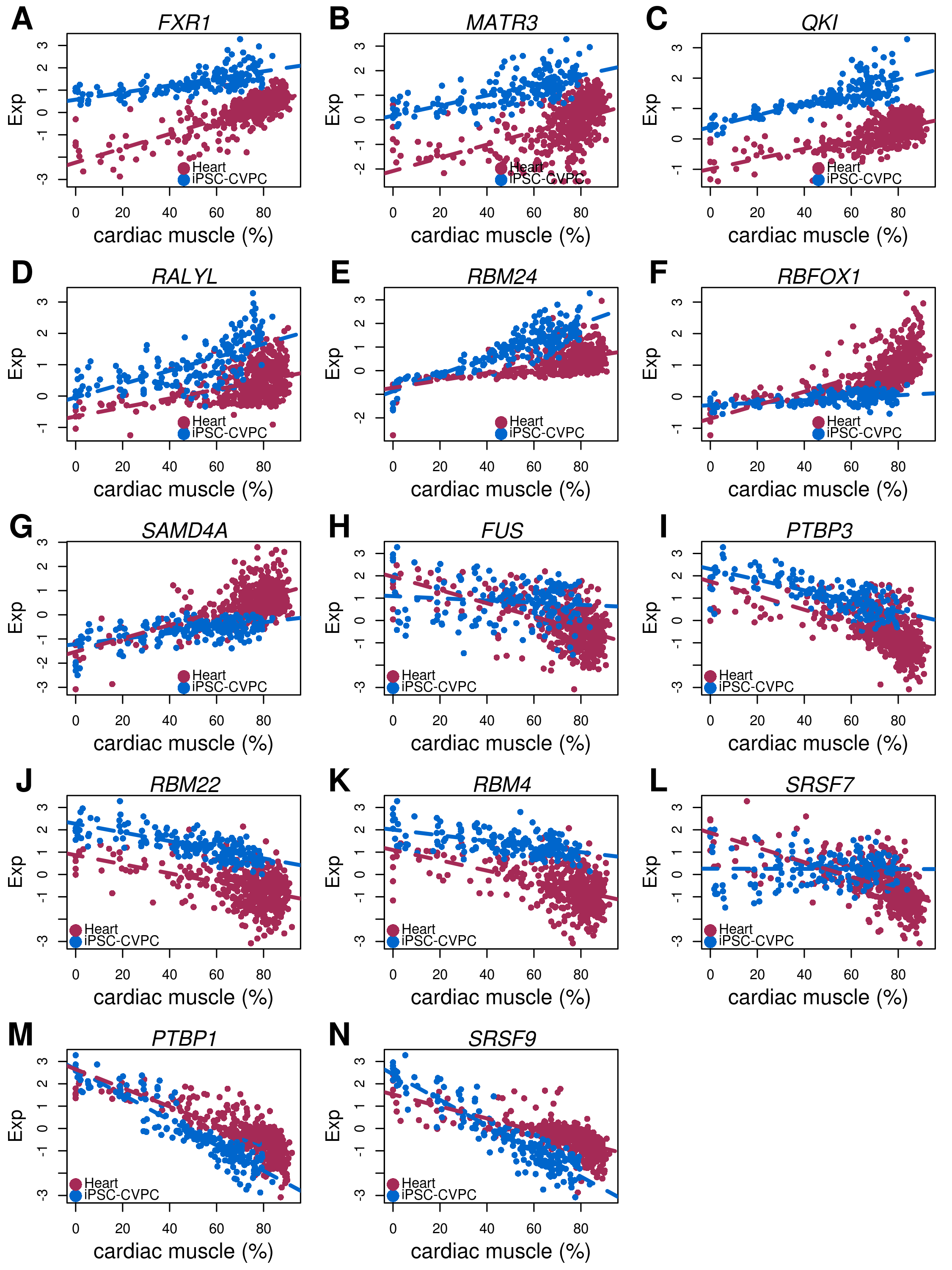

Supplement: S16 Fig — Scatterplots showing cardiac muscle proportions (X axis) and normalized expression in iPSC-CVPC and adult heart (Y axis) for the RBPs that were both differentially expressed between iPSC-CVPC and adult heart (FDR < 0.05) and positively associated with at least one cell type (effect size > 0 and FDR < 0.05). Dashed lines represent regression lines calculated on each CVS tissue. (TIFF) [file pcbi.1009918.s016.tiff]
