# Supplementary material for: In heart failure reactivation of RNA-binding proteins is associated with the expression of 1,523 fetal-specific isoforms
Source: PLoS Comput Biol. 2022 Feb 28;18(2):e1009918. doi: 10.1371/journal.pcbi.1009918 (PMC8912908; doi:10.1371/journal.pcbi.1009918)
Supplement: Attachment — Submitted filename: dantonio_response.pdf [file pcbi.1009918.s036.pdf]

We thank the reviewers for their thoughtful comments. We have incorporated the comments by the three Reviewers and rewritten the Methods section. Additionally, we have made several improvements to the manuscript text and figures that were not requested by a specific reviewer but which we felt could be clearer as we responded to reviewers' comments. Overall, we feel that these changes have resulted in a much-improved paper.

Reviewer #1

The authors integrated 996 RNA-seq samples from heart failure, heathy heart and arteria as well as fetal-like CVS tissues and revealed that RNA-binding proteins are highly overexpressed in fetal-like compared with health adult and are reactivated in heart failure. Also, they showed that overexpression of RBPs in heart failure is associated with a transcriptome-wide isoform switch, which could be used as novel therapeutics for heart failure.

Overall, the manuscript is well written and easy to follow. The description of the methods and data sources is very clear. All calculations and use of statistics throughout the manuscript were properly carried out. I have no major concerns of the approach or the conclusions.

Minor Comments:

Figure 6B: The colors of LVAD are a little confused, could you change the colors of the arrows?

We thank the reviewer for pointing this out and have changed the color of the heart failure arrows to dark green.

Figure 5D-G: Could you add the p-values on the figures so that readers could easily catch the information by only looking at the figures.

We have added p-values to Figure 5D-G and added the following to its legend: "P-values represent the association between the use of each isoform and the cell type proportions (cardiac muscle in panels D and E, cardiac neuron in panel F)" and "P-value represents the association between the *PTBP1* expression and the cardiac neuron proportion".

Reviewer #2:

This research work focuses on investigating differential gene expressions and alternative splicings related to heart failure. Bulk RNAseq data of a health patient cohort matched with samples of induced pluripotent stem cells from cardiovascular progenitor cells (iPSC-CVPC) were generated and analyzed to identify differential expressed genes and isoforms of alternative use between iPSC-CVPC samples and adult samples. The analysis revealed significant differential expressions with stage specific isoforms in RNA binding proteins (RBPs). Further comparison with another cohort of heart failure patient data reveals fetal-like expressions of RBPs and informs in these heart failure samples. While the study uses a novel approach to investigate an important research question, the overall study design and the data analysis seem to need some more improvements as outlined below.

Major:

1. One strength of the study is the integration of multiple sets of samples and datasets to make the investigation possible. However, one caveat is the possible technical biases and batch effects among the gene expression datasets. The sample separations in Figure 1(A) could be due to tissue/developmental stage specificity or technical bias from different preparation of iPSC-CVPC and iPSCORE samples. The linear regression model with additive factors cannot distinguish tissue type from data/sample batch. It might be a good idea to utilize the paired samples between iPSC-CVPCs and adult hear/arteria samples to improve the reliability of the analysis. Given the large number of differentially expressed genes detected in the analysis, the results in Figure 1 and Figure 2 should be at least further validated with the paired samples. Without distinguishing tissue type and such possible technical bias or batch effect, the results are not convincing. This is a major limitation of the current analysis.

We agree with the Reviewer that obtaining paired fetal-like iPSC-CVPC and adult cardiac samples would be optimal to minimize technical biases and batch effects, but this is not possible as these samples do not exist. The iPSCs in iPSCORE were generated from healthy individuals and then differentiated into iPSC-CVPCs. The adult CVS samples used in our study were obtained from GTEx. We apologize for the confusion and have edited the methods to make this point clearer (see response to comment 4 below).

We understand the reviewer's concern about batch effects. To remove false positives due to batch effects we have filtered our data in the following manner:

1. We eliminated all the genes and isoforms whose expression is strongly associated with read length, which is the main technical difference between the iPSCORE and GTEx collections, as described in the "Integration of iPSCORE and GTEx datasets" section in the Methods;

2. To perform differential expression, we used the following sequencing-associated features, which may result in strong technical biases, as covariates: 1) normalized number of RNA-seq reads; 2) % of reads mapping to autosomes or sex chromosome; and 3) % of mitochondrial reads.
3. Because differences in the proportion of cell types between samples could result in differential expression differences that are not due to developmental stage, we used CIBERSORT to estimate cellular composition and repeated the differential expression analysis using ridge regression with cell proportions as co-variates (Figure 4). Ridge regression minimizes the effects of cell type proportions as a confounding factor and accounts for the fact that the cell type proportion covariates are not independent. These data show that the transcriptomic differences between the two adult CVS tissues are largely due to different cell type proportions, whereas cellular composition does not play a major role in the observed transcriptomic differences between fetal-like iPSC-CVPC and adult CVS tissues.

Furthermore, we used the results of previous small-scale studies that investigated the changes in gene expression and/or isoform switching between fetal and adult cardiac tissues to validate our findings. The following examples have been better emphasized in the Results section and are now also described in the Discussion.

1. RBPs known to be involved in fetal cardiac development (including *CELF1*, *HNRNPL*, *RBFOX2* and *RBM24*) are overexpressed in fetal-like iPSC-CVPCs. Additional RBPs overexpressed in iPSC-CVPC are *TAF15*, involved in cell proliferation; *IGF2BP1*, which regulates fetal hemoglobin alternative splicing; *PABPC1*, which regulates translation and cardiomyocyte growth; and *RBM4*, which regulates striated muscle differentiation.
2. RBPs with known adult functions (*MBNL1* and *RBFOX1*) are overexpressed in adult CVS tissues;
3. *ZFP36* was the only RBP overexpressed in both adult heart and arteria, compared with iPSC-CVPC, consistent with its involvement in the inhibition of the immune and inflammatory responses highly expressed in atherosclerotic lesions, where it controls inflammatory response by inhibiting the expression of proinflammatory transcripts;
4. We confirmed that the major cardiac development markers known to undergo isoform switching between fetal and adult heart (*SCN5A*, *TNNT2*, *ABLIM1* and *TTN*) express the appropriate fetal- and adult-specific isoforms respectively in fetal-like iPSC-CVPC and adult CVS tissues;
5. We used CIBERSORT to determine the cell type proportions in each of the 966 cardiac samples and validated the associations between gene expression and cell type proportions using gene set enrichment analysis (Table S14);
6. To further validate the associations between gene expression levels and cell type proportions we determined the correlation between the effect sizes that we determined and those obtained from two independent single cell RNA-seq experiments [1, 2];
7. Most importantly, we observe 3,358 differentially expressed genes between the two adult CVS tissues (heart and arteria). Adult CVS samples were obtained from the same individuals and treated using the same protocols to minimize biases within the GTEx collection [3, 4]. It is not surprising that thousands of genes are differentially expressed between two adult tissues composed of the same cell types in different proportions. Indeed, using ridge regression, which takes the cell type proportions into account, the number of differentially expressed genes between

the two adult CVS tissues drops to 218. Conversely, 5,919 and 3,429 genes were differentially expressed between iPSC-CVPC and adult heart and arteria, respectively, when adjusting for the cell type proportions, suggesting that the same cell types at the two developmental stages are associated with different transcriptomic properties.

Thus, while we cannot exclude that some of our results are due to technical biases, most of the results likely reflect biological differences between fetal-like iPSC-CVPC and adult CVS tissues.

To address the Reviewer's concern we have modified the following:

- Results section "RNA binding proteins show extensive expression differences between fetal-like and adult CVS tissues": "We next conducted pairwise gene expression comparisons of the three CVS tissues (fetal-like iPSC-CVPCs, adult heart, adult arteria) using linear regression and adjusting for possible sources of technical bias"; "Because thousands of genes were differentially expressed between the iPSCORE fetal-like iPSC-CVPC and the GTEx adult CVS tissues, we investigated their function to confirm that our findings reflect biological differences, rather than technical artifacts"; and "Our functional enrichment analysis shows that iPSC-CVPC have more tissue-specific and overexpressed RBPs than either adult heart or adult arteria, and while there is a greater number of overexpressed RBPs in adult arteria compared with adult heart, the two adult CVS tissues have similar numbers of tissue-specific RBPs expressed, suggesting that RBP overexpression is a feature associated with fetal heart development";

- Results section "Large-scale isoform switching occurs between fetal-like and adult CVS developmental stages": "To confirm that the isoform usage differences between fetal-like iPSC-CVPC and adult heart and arteria were biologically relevant, we tested if we could capture the isoform switching of four cardiac genes known to have well-established developmental stage-specific isoforms (*SCN5A*, *TNNT2*, *ABLIM1* and *TTN*) and for all these genes, we found the expected associations between isoform expression and CVS developmental stage (Figure S7)".

2. Given the more biological focus of this study, it is somewhat expected that the findings should be further validated with additional laboratory experiments. For example, the alternative splicing and differential expressions in some specific RBPs shown in Figure 2 can be further validated with PCR or protein staining. Without such validation (even a small scale one), the findings still remain quite hypothetical.

We thank the reviewer for this comment and apologize for not adequately describing the existing data that supports the biological relevance of our findings. In the updated manuscript, we provide additional support from previous studies that have characterized the expression differences of specific RBPs and isoforms between fetal and adult cardiac samples. Our results confirm these previously validated examples, therefore we believe further small-scale experimental validations would be redundant. Specifically, we confirm the following associations:

1. Section "RNA binding proteins show extensive expression differences between fetal-like and adult CVS tissues":

a. RBPs known to be involved in fetal cardiac development (including *RBFOX2*, *HNRNPL*, *RBM24* and *CELF1*) and with adult functions (*RBFOX1* and *MBNL1*) [5-11] were respectively overexpressed in the iPSC-CVPC and adult CVS tissues (Figure 1D, Figure S6)
b. Additional RBPs overexpressed in iPSC-CVPCs included: *TAF15*, involved in cell proliferation [12] (Figure 1E, Table S3); *IGF2BP1*, which regulates fetal hemoglobin alternative splicing [13] (Figure 1F); *PABPC1*, which regulates translation and cardiomyocyte growth [14] (Figure 1G); and *RBM4*, which regulates striated muscle differentiation [15] (Figure 1H).
c. *RBM20*, which is associated with dilated cardiomyopathy [16-18], was overexpressed in both iPSC-CVPC and adult heart compared with adult arteria (respectively, $p = 1.5 \times 10^{-30}$ and $p = 1.3 \times 10^{-55}$, Figure 1I), consistent with its predominant expression in striated muscle [19].
d. The only RBP that was significantly overexpressed in both adult heart and adult arteria, compared with fetal-like iPSC-CVPC, was *ZFP36* (heart: $\log_2$ ratio = -2.8, $p = 3.6 \times 10^{-62}$; and arteria: $\log_2$ ratio = -4.7, $p = 4.1 \times 10^{-78}$, Figure 1J). Consistent with its adult-specific expression, this gene is involved in the inhibition of the immune and inflammatory responses highly expressed in atherosclerotic lesions, where it controls inflammatory response by inhibiting the expression of proinflammatory transcripts [20, 21].

2. Section "Large-scale isoform switching occurs between fetal-like and adult CVS developmental stages":
    a. We confirmed the presence of cardiac developmental stage-specific isoforms for well-known cardiac markers (*SCN5A*, *TNNT2*, *ABLIM1* and *TTN*, Figure S7);
    b. We discovered that 38 RBPs (31.1% of expressed RBPs) had isoforms with developmental stage-specific expression, including RBPs with known cardiac functions such as *MBNL1*, *FXR1*, *HNRNPM* and *FMR1* (Figure 2B-E).
3. Section "Differential RBP expression associated with developmental stage- and cell type-specific usage of isoforms":
    a. Known functions of several of the RBPs positively associated with cardiac muscle proportion are consistent with these observations: 1) *MATR3* is strongly expressed in the mouse heart, limb and brain during embryonic development, but is not expressed in their adult counterparts, and its disruption is associated with multiple congenital heart defects [22]; 2) *QKI* is involved in the regulation of apoptosis in cardiac muscle cells [23] and regulates cardiac myofibrillogenesis [24]; 3) *RBM24* regulates muscle-specific alternative splicing of genes involved in the sarcomere assembly and integrity during cardiac development, including *ACTN2*, *TTN* and *MYH10* [25, 26]; and 4) *SAMD4A* plays important functions in both embryonic development and muscle homeostasis [27, 28].
    b. We validated the spatiotemporal context of the association between a RBP and the alternative splicing of its known target gene, *PTBP1* and *EXOC7* [29].

Given the numerous genes and isoforms that we identified as differentially expressed that have been validated using literature sources, we believe that small-scale laboratory experiments to further validate one or two more genes and/or

isoforms of the thousands that are differentially expressed would not substantially add to the overall strength of the results presented in this manuscript.

3. In the results on page 6 and Figure 2, the analysis of isoform only focuses on the proportions. It is also important to show differential absolute expression or the genes/isoforms, given the selected examples of RBPs in Figure 1 and Figure 2 are quite different. When the gene expression is lower, the isoform quantification is often less reliable. It is important to also visualize the expressions in addition to the proportions.

We performed differential isoform use using transcript fraction, rather than isoform TPM, as this is one of the best practices of *RSEM* as described in the original Li and Dewey paper [30], "The transcript fraction measure is preferred over the popular RPKM and FPKM measures because it is independent of the mean expressed transcript length and is thus more comparable across samples and species". We considered a gene as expressed if it had TPM ≥ 1 in at least 10% of the samples. Across all expressed genes, we analyzed the expression of isoforms with at least 10% usage in at least 10% of the samples. Only isoforms of genes with at least two expressed isoforms were considered (see Methods section "Integrated analysis of iPSCORE and GTEx RNA-seq datasets"). We have modified the Methods section "Integrated analysis of iPSCORE and GTEx RNA-seq datasets": "We used isoform usage %, rather than isoform TPM, because isoform use is independent of the transcript length and therefore more comparable across samples [30]. This resulted in a total of 20,393 genes and 38,271 isoforms (corresponding to 10,686 genes) used in the differential expression analysis".

4. The organization of the article especially the method section can still be improved. Many different analyses are described with very sparse detail. There are three major analysis in the manuscript, differential gene expressions and alternative splicing analysis, functional motif/exon enrichment, and cell type decomposition. The specific analysis steps can be better integrated and explained together for each of the major analysis.

We thank the reviewer for this comment and agree that the Methods sections should be reorganized to make all the analyses that we performed clearer for the reader. As suggested by Reviewers 2 and 3, we have reorganized the Methods into six sections:

1. "RNA-seq data", which includes a description of the iPSCORE and GTEx datasets, as well as their integration;
2. "Transcriptome-wide gene expression and isoform usage differences between CVS tissues", which describes the differences between iPSC-CVPC, adult heart and adult arteria;
3. "RBP and alternative splicing analyses", which includes the RBP motif and exon enrichment analyses;
4. "CVS tissue cellular deconvolution and validation of cell-type proportions", which includes the cell type deconvolution analysis;
5. "Developmental stage- and cell-type-associated gene expression and isoform usage", which describes the ridge regression analysis to examine whether the transcriptional differences between the fetal-like iPSC-CVPC and two adult CVS tissues were due to expression of developmental stage-specific genes and isoforms;

6. “Comparing heart failure with fetal-like and adult heart transcriptomes”, which describes the similarities between the fetal-like iPSC-CVPC and heart failure transcriptomes.

Minor:

1. In the results on top of page 5, it is not stated how many samples are determined heart and how many are arteria. Neither in Figure 1 and Figure S2.

We have included this information at the beginning of the Results section “RNA binding proteins show extensive expression differences between fetal-like and adult CVS tissues”: “To examine global transcriptome-wide gene expression differences between fetal-like iPSC-CVPC and adult CVS tissues we performed dimensionality reduction and clustering of the 966 bulk RNA-seq samples (180 iPSC-CVPCs, 227 aorta, 125 coronary arteries, 196 atrial appendages and 238 left ventricles; Figure S1, Table S1, Table S2)”.

2. The results in Figure 3B and Figure S8 are confusing. Especially, it seems the eCLIP peak enrichment/depletion switches when different log2 thresholds are used to pick the candidate isoforms of each gene. More explanation and justification are needed to support the conclusion.

We thank the Reviewer for pointing out that the data shown in Figure 3B and Figure S8 was confusing. In our original submission, the heatmap on these figures showed, as positive thresholds on the X axis, the enrichment values for iPSC-CVPC-specific isoforms to overlap each eCLIP peaks of each RBP, whereas negative thresholds represented the enrichments for adult heart-specific isoforms. We realize that this representation is confusing, therefore we changed it to a more straightforward plot by displaying lines representing each eCLIP experiment and tissue, as well as the average across all eCLIP experiments for each tissue. We think that this representation better clarifies our conclusions that iPSC-CVPC-specific isoforms are more likely to overlap eCLIP peaks than adult heart- and arteria-specific isoforms.

We have updated Figure 3B and Figure S8 and their legends. We have also modified the methods section and Table S8 legend to clarify the results shown in the two figures.

3. It is unclear if all the analyses are done after the integration of iPSCORE and GTEx datasets as described on page 14. This step is unnecessary for the analysis between IPSC-CVPC and iPSCORE samples. Please clarify.

We apologize for the confusion. We performed RNA-seq on the iPSC-CVPC samples included in the iPSCORE collection, which we derived and analyzed in previous studies [1, 31]. To limit biases due to differences in RNA-seq pipelines, we obtained FASTQ files from GTEx and processed them using the same pipeline. Next, we integrated all the 966 samples and performed all the downstream analyses described in this study.

To clarify this and other concerns by the Reviewers, we have reorganized the Methods section (see response to comment 4 above). We have also added the following to the "Integrated analysis of iPSCORE and GTEx RNA-seq datasets" section in the Methods: "All the analyses described in this study were performed on the gene expression and isoform use data integrated between the iPSCORE and GTEx collections".

4. The step of removing genes and isoforms for the correction of read length bias is somewhat strange. Isn't read length bias systematic rather than gene or isoform specific? The correction does not seem to accomplish the goal.

As the Reviewer suggested in comment 1, obtaining and analyzing data from multiple sources may result in batch effects. Although our validation analyses described above in response to Comments 1 and 2 show that the differences in gene expression and isoform usage have biological relevance, using RNA-seq with different read lengths could result in some biases. The read lengths for the iPSCORE samples was 150 bp paired-end while for the GTEx samples the read length was 75 bp paired-end. Therefore, we decided to use a very stringent approach: we reanalyzed a subset of the iPSC-CVPC samples by hard-clipping their read length to 75 bp and tested the differences in gene expression and isoform usage between the same samples treated as 150 bp paired-end and as 75 bp paired end. We identified 299 genes and 8,999 isoforms whose expression levels are influenced by read length, which we removed from our downstream analyses, because otherwise it would have been difficult to distinguish if the differential expression between iPSC-CVPC (iPSCORE) and adult heart (GTEx) is due to biases related to the different read lengths or due to real biological differences or simply because of between iPSCORE and GTEx samples.

We have improved the description of this source of batch effects in the Methods section "Addressing potential batch effects" and in Table S19 legend.

5. In "Defining RBPs", are the RBPs already given from the sources or identified by matching the PWMs against the genes in this study?

We obtained PWMs from the three sources and then we removed all the RBPs that were not expressed in the CVS tissues. We have improved the description of this in the Methods. Specifically we describe the following: "We obtained the position weight matrix (PWM) of RBPs included the RNA binding gene set (GO:0003723) from three sources: 1) RBPmap V.1.1 [32]; 2) ENCODE [33]; and 3) CISBP-RNA V.0.6 [34]. For the analyses described in the manuscript, we considered only the 122 RBPs that both had a known motif (262 PWMs in total) obtained from these sources and were expressed in the 966 cardiac samples".

6. Please provide more detail (statistics) of eCLIP peak data. How was the peak called?

Peaks were called in the original ENCODE eCLIP manuscript by Van Norstrand et al. [33].We only obtained BED files with the peaks called by eCLIP. We describe that we downloaded processed data (BED files with eCLIP peaks) in the improved Methods section “Enrichment of CVS developmental stage-specific isoforms for RBP binding sites”:

We obtained *in vitro* binding activity of 39 RBPs using enhanced CLIP (eCLIP, 59 experiments) from a recent ENCODE study [33]. One RBP (*MATR3*) was not expressed in CVS tissues and was removed from further analyses. We downloaded BED files corresponding to biologically reproducible eCLIP peaks and intersected them with the genomic coordinates of the loci encoding each gene (defined as the start-end coordinates obtained from Gencode) expressed in the 966 cardiac samples. To test whether a CVS tissue was enriched for having active RBP binding sites, we performed pairwise comparisons (iPSC-CVPC vs. adult heart, iPSC-CVPC vs. adult arteria and as a control adult heart vs. adult arteria) using the following approach:

1) For each pairwise comparison, we obtained the list of isoforms overexpressed in tissue 1 and those overexpressed in tissue 2, and converted them to their associated genomic coordinates. To examine overexpressed isoforms, we used 13 filtering thresholds, from $\log_2$ ratio = 0 (the least stringent, including all the isoforms that had a significant p-value after FDR correction in Table S5) to $\log_2$ ratio > 6 (Figure 3B, Figure S8), in 0.5 increments.
2) As background, we used all the genes that did not have any differentially expressed isoforms in the pairwise comparison between tissue 1 and tissue 2.
3) We intersected the three lists of genomic coordinates with the coordinates of eCLIP peaks for each eCLIP experiment.
4) We compared the fraction of genes associated with tissue 1 against background using Fisher’s exact test (*fisher.test* in R); likewise, we compared the fraction of genes associated with tissue 2 against background.

Reviewer #3:

This manuscript performed systematic analyses of published datasets (GTEx, iPSCORE, heart failure bulk RNA-seq, Tabula Muris) and investigated the role of RNA binding protein and splicing in CVS development and heart failure. I found that the manuscript is generally interesting, and the analyses are comprehensive. Still, I would prefer more details/clarifications of the methods and results to better facilitate readers' understanding of this manuscript.

1. In the abstract, the authors state that 'Comparison of the expression profiles revealed that RNA-binding proteins (RBPs) are highly overexpressed in fetal-like compared with healthy adult and are reactivated in heart failure, which results in expression of thousands fetal-specific isoforms' I am not sure whether the manuscript has enough data to support the causality.

We agree that our results do not show causality and have changed this sentence to "Comparison of the expression profiles revealed that 33 RNA-binding proteins (RBPs) are highly overexpressed in fetal-like compared with healthy adult, of which 14 are reactivated in heart failure and are associated with changes in the expression of thousands fetal-specific isoforms. Of note, isoforms for 20 different RBPs were among those that reverted in heart failure to the fetal-like expression pattern".

2. More detailed information should be provided regarding how the differential expression analysis and the isoform analysis were performed. The methods section should provide more information regarding this.

We agree that the Methods section was not well-organized. Following the suggestions by Reviewer 2 (comment 4), we have rewritten the Methods and divided them into six sections: "RNA-seq data", "Transcriptome-wide gene expression and isoform usage differences between CVS tissues", "RBP and alternative splicing analyses", "CVS tissue cellular deconvolution and validation of cell-type proportions", "Developmental-stage- and cell-type-associated gene expression and isoform usage" and "Comparing heart failure with fetal-like and adult heart transcriptomes". We have also provided more detailed descriptions in our Methods.

3. In Figure 3A-B, are there differences between the 33 RBPs which were overexpressed in iPSC-CVPC and the six which were not differentially expressed?

We did not observe significant differences between the 33 RBPs overexpressed in iPSC-CVPC and the six that were not differentially expressed in the enrichment of their associated eCLIP peaks for overlapping iPSC-CVPC-specific or adult heart-specific isoforms. The take-home message from this figure is that, at all thresholds, iPSC-CVPC-specific isoforms are more likely to overlap eCLIP peaks than adult CVS-specific isoforms, suggesting that RBPs may play a larger role controlling the expression of fetal-specific isoforms than adult-specific isoforms.

However, we realize that the representation of the results shown in Figure 3B was confusing (as pointed out on minor comment 2 by Reviewer 2), and have changed both Figure 3B and Figure S8.

4. Interestingly, the heart failure sample has fetal-like expression patterns. How about other types of cardiovascular disease? I assume this observation will be more relevant to the disease related to cardiovascular development versus the others.

The Reviewer has a great question which unfortunately does not have a straightforward answer. A variety of developmental disorders (including arrhythmias, congenital heart and cardiac valve defects) and adult cardiovascular diseases (coronary artery disease, myocardial infarction, hypertension, myocarditis, dilated cardiomyopathy) may result in heart failure, which is a general condition that develops when the heart does not pump enough blood for a body's needs (https://www.nhlbi.nih.gov/health-topics/heart-failure; https://www.mayoclinic.org/diseases-conditions/heart-failure/symptoms-causes/syc-20373142). The heart failure samples analyzed in this manuscript were derived from ischemic cardiomyopathy (ICM) and non-ischemic cardiomyopathy (NICM) before and after mechanical circulatory support with a left ventricular assist device (LVAD) [35]. We used this dataset for multiple reasons:

1. It is one of the largest heart failure RNA-seq datasets available (30 samples from 15 individuals);
2. RNA-seq was paired-end 100 bp, which is similar to both the iPSC-CVPCs (paired-end 150 bp) and GTEx (paired-end 75 bp), thereby limiting the potential technical biases due to different sequencing methods;
3. More importantly, this dataset includes both pre- and post-LVAD paired samples, which allowed us to characterize the trajectory of each sample from heart failure (pre-LVAD) to healthier heart (post-LVAD).

While we agree with the Reviewer that adding samples with heart failure caused by other cardiac diseases would greatly expand our knowledge of the extent to which the cardiac transcriptome reverts to fetal-like, unfortunately, we could not find other relevant datasets that are technically similar to the ones used in this study.

5. It is unclear how the motif analysis for the 122 RBPs (Figure S9) was conducted. Please provide more information to clarify this analysis. Was the analysis performed on all the iPSC-CVPC-specific, adult heart-specific, and adult arteria-specific exons using the motif of the 122 RBPs? Which motifs (source) were used, and how the motif search was performed?

We have reorganized and re-written the Methods Section, to address the Reviewer's comment.

In the section "RBPs with known motifs": "We obtained the position weight matrix (PWM) of RBPs included the RNA binding gene set (GO:0003723) from three sources: 1) RBPmap V.1.1 [32]; 2) ENCODE [33]; and 3) CISBP-RNA V.0.6 [34]. For the analyses described in the manuscript, we considered only the 122 RBPs that both had a known motif (262 PWMs in total) obtained from these sources and were expressed in the 966 cardiac samples". To make this clearer, we have also rewritten the Result section "iPSC-CVPC-specific exons are enriched for RBP binding and canonical splice sites" and the Method section "Enrichment of CVS developmental stage-specific exons for RBP motifs".

Have the authors made all data and (if applicable) computational code underlying the findings in their manuscript fully available?

The PLOS Data policy requires authors to make all data and code underlying the findings described in their manuscript fully available without restriction, with rare exception (please refer to the Data Availability Statement in the manuscript PDF file). The data and code should be provided as part of the manuscript or its supporting information, or deposited to a public repository. For example, in addition to summary statistics, the data points behind means, medians and variance measures should be available. If there are restrictions on publicly sharing data or code —e.g. participant privacy or use of data from a third party—those must be specified.

Reviewer #1: Yes

Reviewer #2: Yes

Reviewer #3: No: Data are available as suggested by the authors, but codes are not (not sure whether it is a must for PLOS computational biology)

All data supporting the results described in this manuscript has been made available. We did not generate new code, but we did create custom R scripts to process the data and generate the figures and tables for this manuscript. Although we have not deposited these scripts, we are happy to share them with interested readers. We have added the following to the Data availability section: “R scripts to process the data and generate the figures and tables for this manuscript are available upon request”.

References:

1. D'Antonio-Chronowska A, Donovan MKR, Young Greenwald WW, Nguyen JP, Fujita K, Hashem S, et al. Association of Human iPSC Gene Signatures and X Chromosome Dosage with Two Distinct Cardiac Differentiation Trajectories. Stem Cell Reports. 2019;13(5):924-38. Epub 2019/11/02. doi: 10.1016/j.stemcr.2019.09.011. PubMed PMID: 31668852; PubMed Central PMCID: PMCPMC6895695.
2. Tucker NR, Chaffin M, Fleming SJ, Hall AW, Parsons VA, Bedi KC, Jr., et al. Transcriptional and Cellular Diversity of the Human Heart. Circulation. 2020. Epub 2020/05/15. doi: 10.1161/CIRCULATIONAHA.119.045401. PubMed PMID: 32403949.
3. Consortium GT. The GTEx Consortium atlas of genetic regulatory effects across human tissues. Science. 2020;369(6509):1318-30. Epub 2020/09/12. doi: 10.1126/science.aaz1776. PubMed PMID: 32913098; PubMed Central PMCID: PMCPMC7737656.
4. Consortium GT, Laboratory DA, Coordinating Center -Analysis Working G, Statistical Methods groups-Analysis Working G, Enhancing Gg, Fund NIHC, et al. Genetic effects on gene expression across human tissues. Nature. 2017;550(7675):204-13. Epub 2017/10/13. doi: 10.1038/nature24277. PubMed PMID: 29022597; PubMed Central PMCID: PMCPMC5776756.
5. Kalsotra A, Xiao X, Ward AJ, Castle JC, Johnson JM, Burge CB, et al. A postnatal switch of CELF and MBNL proteins reprograms alternative splicing in the developing heart. Proc Natl Acad Sci U S A. 2008;105(51):20333-8. Epub 2008/12/17. doi: 10.1073/pnas.0809045105. PubMed PMID: 19075228; PubMed Central PMCID: PMCPMC2629332.
6. Gao C, Ren S, Lee JH, Qiu J, Chapski DJ, Rau CD, et al. RBFox1-mediated RNA splicing regulates cardiac hypertrophy and heart failure. J Clin Invest. 2016;126(1):195-206. Epub 2015/12/01. doi: 10.1172/JCI84015. PubMed PMID: 26619120; PubMed Central PMCID: PMCPMC4701548.
7. Gazzara MR, Mallory MJ, Roytenberg R, Lindberg JP, Jha A, Lynch KW, et al. Ancient antagonism between CELF and RBFOX families tunes mRNA splicing outcomes. Genome Res. 2017;27(8):1360-70. Epub 2017/05/18. doi: 10.1101/gr.220517.117. PubMed PMID: 28512194; PubMed Central PMCID: PMCPMC5538552.
8. Zhang M, Zhang Y, Xu E, Mohibi S, de Anda DM, Jiang Y, et al. Rbm24, a target of p53, is necessary for proper expression of p53 and heart development. Cell Death Differ. 2018;25(6):1118-30. Epub 2018/01/24. doi: 10.1038/s41418-017-0029-8. PubMed PMID: 29358667; PubMed Central PMCID: PMCPMC5988652.
9. Zhao Y, Zhou J, He L, Li Y, Yuan J, Sun K, et al. MyoD induced enhancer RNA interacts with hnRNPL to activate target gene transcription during myogenic differentiation. Nat Commun. 2019;10(1):5787. Epub 2019/12/21. doi: 10.1038/s41467-019-13598-0. PubMed PMID: 31857580; PubMed Central PMCID: PMCPMC6923398.

10. Loiselle JJ, Sutherland LC. Differential downregulation of Rbm5 and Rbm10 during skeletal and cardiac differentiation. In Vitro Cell Dev Biol Anim. 2014;50(4):331-9. Epub 2013/11/02. doi: 10.1007/s11626-013-9708-z. PubMed PMID: 24178303.
11. Giudice J, Xia Z, Wang ET, Scavuzzo MA, Ward AJ, Kalsotra A, et al. Alternative splicing regulates vesicular trafficking genes in cardiomyocytes during postnatal heart development. Nat Commun. 2014;5:3603. Epub 2014/04/23. doi: 10.1038/ncomms4603. PubMed PMID: 24752171; PubMed Central PMCID: PMCPMC4018662.
12. Ballarino M, Jobert L, Dembele D, de la Grange P, Auboeuf D, Tora L. TAF15 is important for cellular proliferation and regulates the expression of a subset of cell cycle genes through miRNAs. Oncogene. 2013;32(39):4646-55. Epub 2012/11/07. doi: 10.1038/onc.2012.490. PubMed PMID: 23128393.
13. de Vasconcellos JF, Tumburu L, Byrnes C, Lee YT, Xu PC, Li M, et al. IGF2BP1 overexpression causes fetal-like hemoglobin expression patterns in cultured human adult erythroblasts. Proc Natl Acad Sci U S A. 2017;114(28):E5664-E72. Epub 2017/06/28. doi: 10.1073/pnas.1609552114. PubMed PMID: 28652347; PubMed Central PMCID: PMCPMC5514697.
14. Chorghade S, Seimetz J, Emmons R, Yang J, Bresson SM, Lisio M, et al. Poly(A) tail length regulates PABPC1 expression to tune translation in the heart. Elife. 2017;6. Epub 2017/06/28. doi: 10.7554/eLife.24139. PubMed PMID: 28653618; PubMed Central PMCID: PMCPMC5487213.
15. Lin JC, Tarn WY. RNA-binding motif protein 4 translocates to cytoplasmic granules and suppresses translation via argonaute2 during muscle cell differentiation. J Biol Chem. 2009;284(50):34658-65. Epub 2009/10/06. doi: 10.1074/jbc.M109.032946. PubMed PMID: 19801630; PubMed Central PMCID: PMCPMC2787328.
16. van den Hoogenhof MMG, Beqqali A, Amin AS, van der Made I, Aufiero S, Khan MAF, et al. RBM20 Mutations Induce an Arrhythmogenic Dilated Cardiomyopathy Related to Disturbed Calcium Handling. Circulation. 2018;138(13):1330-42. Epub 2018/04/14. doi: 10.1161/CIRCULATIONAHA.117.031947. PubMed PMID: 29650543.
17. Brauch KM, Karst ML, Herron KJ, de Andrade M, Pellikka PA, Rodeheffer RJ, et al. Mutations in ribonucleic acid binding protein gene cause familial dilated cardiomyopathy. J Am Coll Cardiol. 2009;54(10):930-41. Epub 2009/08/29. doi: 10.1016/j.jacc.2009.05.038. PubMed PMID: 19712804; PubMed Central PMCID: PMCPMC2782634.
18. Beqqali A, Bollen IA, Rasmussen TB, van den Hoogenhof MM, van Deutekom HW, Schafer S, et al. A mutation in the glutamate-rich region of RNA-binding motif protein 20 causes dilated cardiomyopathy through missplicing of titin and impaired Frank-Starling mechanism. Cardiovasc Res. 2016;112(1):452-63. Epub 2016/08/09. doi: 10.1093/cvr/cvw192. PubMed PMID: 27496873.
19. Guo W, Schafer S, Greaser ML, Radke MH, Liss M, Govindarajan T, et al. RBM20, a gene for hereditary cardiomyopathy, regulates titin splicing. Nat Med. 2012;18(5):766-73. Epub 2012/04/03. doi: 10.1038/nm.2693. PubMed PMID: 22466703; PubMed Central PMCID: PMCPMC3569865.
20. Zhang H, Taylor WR, Joseph G, Caracciolo V, Gonzales DM, Sidell N, et al. mRNA-binding protein ZFP36 is expressed in atherosclerotic lesions and reduces inflammation in aortic

endothelial cells. Arterioscler Thromb Vasc Biol. 2013;33(6):1212-20. Epub 2013/04/06. doi: 10.1161/ATVBAHA.113.301496. PubMed PMID: 23559629; PubMed Central PMCID: PMCPMC3844532.

21. Moore MJ, Blachere NE, Fak JJ, Park CY, Sawicka K, Parveen S, et al. ZFP36 RNA-binding proteins restrain T cell activation and anti-viral immunity. Elife. 2018;7. Epub 2018/06/01. doi: 10.7554/eLife.33057. PubMed PMID: 29848443; PubMed Central PMCID: PMCPMC6033538.

22. Quintero-Rivera F, Xi QJ, Keppler-Noreuil KM, Lee JH, Higgins AW, Anchan RM, et al. MATR3 disruption in human and mouse associated with bicuspid aortic valve, aortic coarctation and patent ductus arteriosus. Hum Mol Genet. 2015;24(8):2375-89. Epub 2015/01/13. doi: 10.1093/hmg/ddv004. PubMed PMID: 25574029; PubMed Central PMCID: PMCPMC4380077.

23. Guo W, Shi X, Liu A, Yang G, Yu F, Zheng Q, et al. RNA binding protein QKI inhibits the ischemia/reperfusion-induced apoptosis in neonatal cardiomyocytes. Cell Physiol Biochem. 2011;28(4):593-602. Epub 2011/12/20. doi: 10.1159/000335755. PubMed PMID: 22178871.

24. Chen X, Liu Y, Xu C, Ba L, Liu Z, Li X, et al. QKI is a critical pre-mRNA alternative splicing regulator of cardiac myofibrillogenesis and contractile function. Nat Commun. 2021;12(1):89. Epub 2021/01/06. doi: 10.1038/s41467-020-20327-5. PubMed PMID: 33397958; PubMed Central PMCID: PMCPMC7782589.

25. Lu SH, Lee KZ, Yeh YC, Pan CY, Hsu PW, Su LY, et al. Alternative Splicing Mediated by RNA-Binding Protein RBM24 Facilitates Cardiac Myofibrillogenesis in a Differentiation Stage-Specific Manner. Circ Res. 2021. Epub 2021/11/25. doi: 10.1161/CIRCRESAHA.121.320080. PubMed PMID: 34816743.

26. Yang J, Hung LH, Licht T, Kostin S, Looso M, Khrameeva E, et al. RBM24 is a major regulator of muscle-specific alternative splicing. Dev Cell. 2014;31(1):87-99. Epub 2014/10/15. doi: 10.1016/j.devcel.2014.08.025. PubMed PMID: 25313962.

27. de Haro M, Al-Ramahi I, Jones KR, Holth JK, Timchenko LT, Botas J. Smaug/SAMD4A restores translational activity of CUGBP1 and suppresses CUG-induced myopathy. PLoS Genet. 2013;9(4):e1003445. Epub 2013/05/03. doi: 10.1371/journal.pgen.1003445. PubMed PMID: 23637619; PubMed Central PMCID: PMCPMC3630084.

28. Niu N, Xiang JF, Yang Q, Wang L, Wei Z, Chen LL, et al. RNA-binding protein SAMD4 regulates skeleton development through translational inhibition of Mig6 expression. Cell Discov. 2017;3:16050. Epub 2017/02/07. doi: 10.1038/celldisc.2016.50. PubMed PMID: 28163927; PubMed Central PMCID: PMCPMC5259697.

29. Georgilis A, Klotz S, Hanley CJ, Herranz N, Weirich B, Morancho B, et al. PTBP1-Mediated Alternative Splicing Regulates the Inflammatory Secretome and the Pro-tumorigenic Effects of Senescent Cells. Cancer Cell. 2018;34(1):85-102 e9. Epub 2018/07/11. doi: 10.1016/j.ccell.2018.06.007. PubMed PMID: 29990503; PubMed Central PMCID: PMCPMC6048363.

30. Li B, Dewey CN. RSEM: accurate transcript quantification from RNA-Seq data with or without a reference genome. BMC Bioinformatics. 2011;12:323. Epub 2011/08/06. doi: 10.1186/1471-2105-12-323. PubMed PMID: 21816040; PubMed Central PMCID: PMCPMC3163565.

31. D'Antonio-Chronowska A, D'Antonio M, Frazer KA. In vitro Differentiation of Human iPSC-derived Cardiovascular Progenitor Cells (iPSC-CVPCs) Bio-Protocol. 2020;10(18). doi: 10.21769/BioProtoc.3755.
32. Paz I, Kosti I, Ares M, Jr., Cline M, Mandel-Gutfreund Y. RBPmap: a web server for mapping binding sites of RNA-binding proteins. Nucleic Acids Res. 2014;42(Web Server issue):W361-7. Epub 2014/05/16. doi: 10.1093/nar/gku406. PubMed PMID: 24829458; PubMed Central PMCID: PMCPMC4086114.
33. Van Nostrand EL, Freese P, Pratt GA, Wang X, Wei X, Xiao R, et al. A large-scale binding and functional map of human RNA-binding proteins. Nature. 2020;583(7818):711-9. Epub 2020/07/31. doi: 10.1038/s41586-020-2077-3. PubMed PMID: 32728246; PubMed Central PMCID: PMCPMC7410833.
34. Ray D, Kazan H, Cook KB, Weirauch MT, Najafabadi HS, Li X, et al. A compendium of RNA-binding motifs for decoding gene regulation. Nature. 2013;499(7457):172-7. Epub 2013/07/13. doi: 10.1038/nature12311. PubMed PMID: 23846655; PubMed Central PMCID: PMCPMC3929597.
35. Yang KC, Yamada KA, Patel AY, Topkara VK, George I, Cheema FH, et al. Deep RNA sequencing reveals dynamic regulation of myocardial noncoding RNAs in failing human heart and remodeling with mechanical circulatory support. Circulation. 2014;129(9):1009-21. Epub 2014/01/17. doi: 10.1161/CIRCULATIONAHA.113.003863. PubMed PMID: 24429688; PubMed Central PMCID: PMCPMC3967509.
