# Supplementary material for: In heart failure reactivation of RNA-binding proteins is associated with the expression of 1,523 fetal-specific isoforms
Source: PLoS Comput Biol. 2022 Feb 28;18(2):e1009918. doi: 10.1371/journal.pcbi.1009918 (PMC8912908; doi:10.1371/journal.pcbi.1009918)
Supplement: Attachment — Submitted filename: dantonio_response.docx [file pcbi.1009918.s037.docx]

Reviewer #2: The authors addressed most of the comments but some concerns regarding the analysis and validation of the differential isoform expressions remain. Specifically:

1. What is the relation between differential gene expression and differential isoform proportion expression? It is understood that only the genes that are considered expressed are used for differential isoform proportion analysis. It also could be true that the isoforms' proportion is more relevant than absolute expression. However, this does not dismiss the importance of analyzing the differential proportions with the context of the absolute gene expression. It will be at least useful to find out whether differential expressed genes also tent to have differential isoform proportion or vice versa etc.? Given the large number of differentially expressed isoforms (19270), more detailed analysis is necessary.

We thank the Reviewer for this insightful comment. It provided us with the opportunity to add an interesting observation to the Results section “Large-scale isoform switching occurs between fetal-like and adult CVS developmental stages”. We tested if genes that had isoforms with differential usage between the fetal-like and adult CVS developmental stages were more likely to be differentially expressed themselves compared with genes whose isoforms did not show differential usage. We observed that differentially expressed genes were indeed more likely to have differentially expressed isoforms, suggesting that differential isoform expression likely contributes to the measurement of differential gene expression.

We have added the following text: “We hypothesized that differential isoform usage may contribute to the measurement of differential gene expression. Therefore, we tested if genes whose isoforms were differentially expressed between the fetal-like iPSC-CVPC and adult heart were more likely to be differentially expressed themselves compared with genes whose isoforms did not show differential usage. We observed that genes that had differential isoform usage were 1.20 times more likely to be differentially expressed (p = 7.3 x 10^-4^, Fisher’s exact test, iPSC-CVPC compared with adult heart) and those that had iPSC-CVPC-specific and/or adult heart-specific isoforms were 1.24 times more likely to be differentially expressed (6.45 x 10^-6^, Figure 2A, Table S5). These results suggest that a significant fraction of the measured differential gene expression is likely due to differences in isoform usage between developmental stages.”

2. It is also understood that there are evidence from the literature supporting the findings in this study. However, it seems none of the evidence is at the isoform level. In Figure 2B-E and Figure S7, the results are from the RNAseq data used for the discovery, which are not validation. Since the discoveries of these isoforms is part of the important contribution of the work, it will be useful to find literature evidence/existing data/new experiments directly support the discoveries. Maybe, some other small scale RNAseq data or protein profiling/staining from other studies can be used for the validation?

We apologize for the miscommunication. The Reviewer is correct that the results presented in Figure 2B-G and S7 are from the data used for the discovery. However, the four genes (*SCN5A*, *TNNT2*, *ABLIM1* and *TTN*) in Figure S7 have isoforms that have been previously published as differentially expressed between fetal and adult cardiac tissues. We included these four genes to validate our observations of differential isoform usage, as the Reviewer requested in the first round of reviews. To make these validations more prominent in our current manuscript, we have: 1) added references to these previous studies in the Introduction (page 4) “We validate the isoform switching of previously described cardiac markers, such as *SCN5A* [23], *TNNT2* [24], *ABLIM1* [25, 26] and *TTN* [4], and show that the expression of thousands of genes and isoforms are associated with both CVS developmental stage and cell type”; 2) describe them in the Results section “Large-scale isoform switching occurs between fetal-like and adult CVS developmental stages” (page 6) “To confirm that the isoform usage differences between fetal-like iPSC-CVPC and adult heart and arteria were biologically relevant, we tested if we could capture the isoform switching of four cardiac genes known to have well-established developmental stage-specific isoforms (*SCN5A*, *TNNT2*, *ABLIM1* and *TTN*) and for all these genes, we found the expected associations between isoform expression and CVS developmental stage (Figure S7)”; and 3) further mention in the Discussion section (page 13) “To validate our observations, we confirmed associations that have been previously reported as small-scale studies, including: 1) major cardiac development markers known to undergo isoform switching between fetal and adult heart (*SCN5A*, *TNNT2*, *ABLIM1* and *TTN*) express the appropriate isoforms respectively in fetal-like iPSC-CVPC and adult CVS tissues”.

Overall, the study is solid but the analysis and the validation of the findings of the isoforms are still lacking.

We hope that, by responding to the comments above, we have strengthened the validations that the Reviewer asks for.
